# Cloning and functional identification of sesquiterpene synthase gene *NjTPS2* in *Nardostachys jatamansi* DC

**DOI:** 10.3389/fpls.2025.1718102

**Published:** 2026-01-12

**Authors:** ZhiYu Hao, LiJun Peng, YueYing Hu, XunJian Wu, YuanYuan Li, JunCheng Zhang, XiaoHui Tang, Jin Pei, Jiang Chen

**Affiliations:** 1 State Key Laboratory of Southwestern Chinese Medicine Resources, Chengdu, Sichuan, China; 2 College of Pharmacy, Chengdu University of Traditional Chinese Medicine, Chengdu, Sichuan, China; 3 Sichuan Academy of Grassland Sciences, Chengdu, China

**Keywords:** clone, functional identification, *Nardostachys jatamansi* DC, NjTPS2, sesquiterpene synthase

## Abstract

**Background:**

*Nardostachys jatamansi* DC is a plant of the Valerianaceae. As an economic crop, it is used in traditional Chinese and Tibetan medicines as well as in spices. The sesquiterpenoids in this plant are the main components that contribute to its medicinal value; however, the current understanding of their biosynthesis pathway remains unclear.

**Methods:**

Volatile components of *N. jatamansi* were detected via metabolomics. Candidate sesquiterpene synthase genes were screened based on transcriptome sequencing results. Quantitative real-time PCR (qRT-PCR) was employed to detect the differential expression of the candidate genes in different tissues of *N. jatamansi*, so as to preliminarily verify the functions of the candidate genes. Finally, the yeast expression system and tobacco transient expression system were utilized to verify the function of the gene *NjTPS2*.

**Results:**

A total of 736 compounds were detected through metabolomic analysis, which were classified into 16 categories. Among them, there were 211 terpenoid compounds and 138 sesquiterpenoid compounds. By performing RNA-seq on *N. jatamansi*, 14.29 Gb of clean data was obtained, and 9 sesquiterpene synthase genes were successfully screened out, which belong to four subfamilies, namely TPSa, TPSb, TPSc, and TPSe/f. The *NjTPS2* gene in the TPSb subfamily was cloned and subjected to bioinformatics analysis. The full length of its CDS (Coding Sequence) is 1212 bp, and domain analysis revealed that it has two core catalytic domains for terpenoids: Terpene synth C and Terpene synth. The results of quantitative real-time PCR (qRT-PCR) showed that *NjTPS2* had the highest expression level in roots, while its expression levels in other tissues were relatively low. Functional verification of *NjTPS2* was conducted using the tobacco transient expression system and yeast expression system, and it was found that this gene could produce sesquiterpenoids.

**Conclusion:**

Our results confirm *NjTPS2* possesses the functional activity of a sesquiterpene synthase. It provides genetic resources for elucidating the molecular mechanism underlying the quality formation of *N. jatamansi*, as well as for the biosynthesis of sesquiterpenoids in *N. jatamansi* and the breeding of high-quality *N. jatamansi* varieties.

## 1 Introduction

*N. jatamansi* is a perennial herb. It is mainly distributed on steep, moist, rocky, and undisturbed grassy slopes at an altitude of 2, 200 to 5, 000 meters in Bhutan, Nepal, India, and the Himalayan region of China (Dhiman and Bhattacharya, 2020). Within China, it is primarily found in Qinghai, Gansu, Yunnan, Sichuan, Tibet, and other regions (Deng et al., 2021). *N. jatamansi* has a long history of application in traditional medicine. It is mentioned in both the ancient Indian Ayurvedic and Unani medical systems (Sahu et al., 2016), and it is also a commonly used clinical variety in the traditional ethnic medicines of Mongolia, Uyghur, Lisu, Naxi and other ethnic groups in China. It is used to treat epilepsy, cholera, boils, leprosy, headaches, palpitations, syncope, as well as diseases of the liver, stomach and heart (Rehman and Ahmad, 2019). Modern pharmacological studies have shown that the pharmacological effects of *N. jatamansi* are mainly attributed to its abundant volatile oil components, especially the sesquiterpenoid components among them, which exhibit various biological activities such as anti-Alzheimer’s disease (Liu et al., 2018), anti-cancer (Sang et al., 2022), anti-inflammatory (Yoon et al., 2018), and anti-oxidant properties (Joshi and Parle, 2006). Due to its special growth environment and large application demand, *N. jatamansi* is currently a critically endangered medicinal plant in China.

Sesquiterpenes are natural terpenoid compounds containing 15 carbon atoms and 3 isoprene units in their molecules. They have skeletal structures such as chains and rings (Das et al., 2024), including types like aristolane (Yu et al., 2016), guaiane (Wang et al., 2008), nardosinane (Ko et al., 2018), eudesmane (Wu et al., 2024), germacrane (Xu and Dickschat, 2020), elemane (Zhang et al., 2024), and bisabolane (Tai et al., 2023). Most of the volatile oils in *N. jatamansi* are small-molecule sesquiterpenes. In 2013, M. L. Liu et al. isolated nardoaristolone B, a 1, 10-dehydroaristolane with a contracted B-ring, from *N. jatamansi* (Liu et al., 2013). The main guaiane-type sesquiterpenes contained in *N. jatamansi* mainly include β-maaliol, aristolan-9-ol, gansongol [1(10)-aristolen-9β-ol], nardostachone [1, 8, 9, 10-tetradehydroaristolan-2-one], debilone, eudesm-11-ene-2, 4α-diol, gansongone, and kanshone A~G. Chai et al. isolated narjatamolide, a guaiane-type sesquiterpene, from the roots and rhizomes of *N. jatamansi* (Chai et al., 2021). Zheng et al. discovered a guaiane-type sesquiterpene, nardoguaianone L, in *N. jatamansi*, and studies have shown that it possesses anti-tumor activity (Zheng et al., 2022). Zhang et al. identified two nardosinane-type sesquiterpenes in *N. jatamansi*, which contain rare 4, 11-epoxy groups (Zhang et al., 2015). L. Shide et al. also identified a nardosinane-type sesquiterpene with this structure from the underground parts of *N. jatamansi* (Shide et al., 1987). Shen et al. isolated six nardosinane-type sesquiterpenes, namely nardosinanones J-N and nardosinanones C, from the roots and rhizomes of *N. jatamansi* (Shen et al., 2017). Currently, the reported sesquiterpenoids from *N. jatamansi* can be classified into three types based on their structures: aristolane-type, guaiane-type, and nardosinane-type (nardostachysane-type).

**Figure 1:**
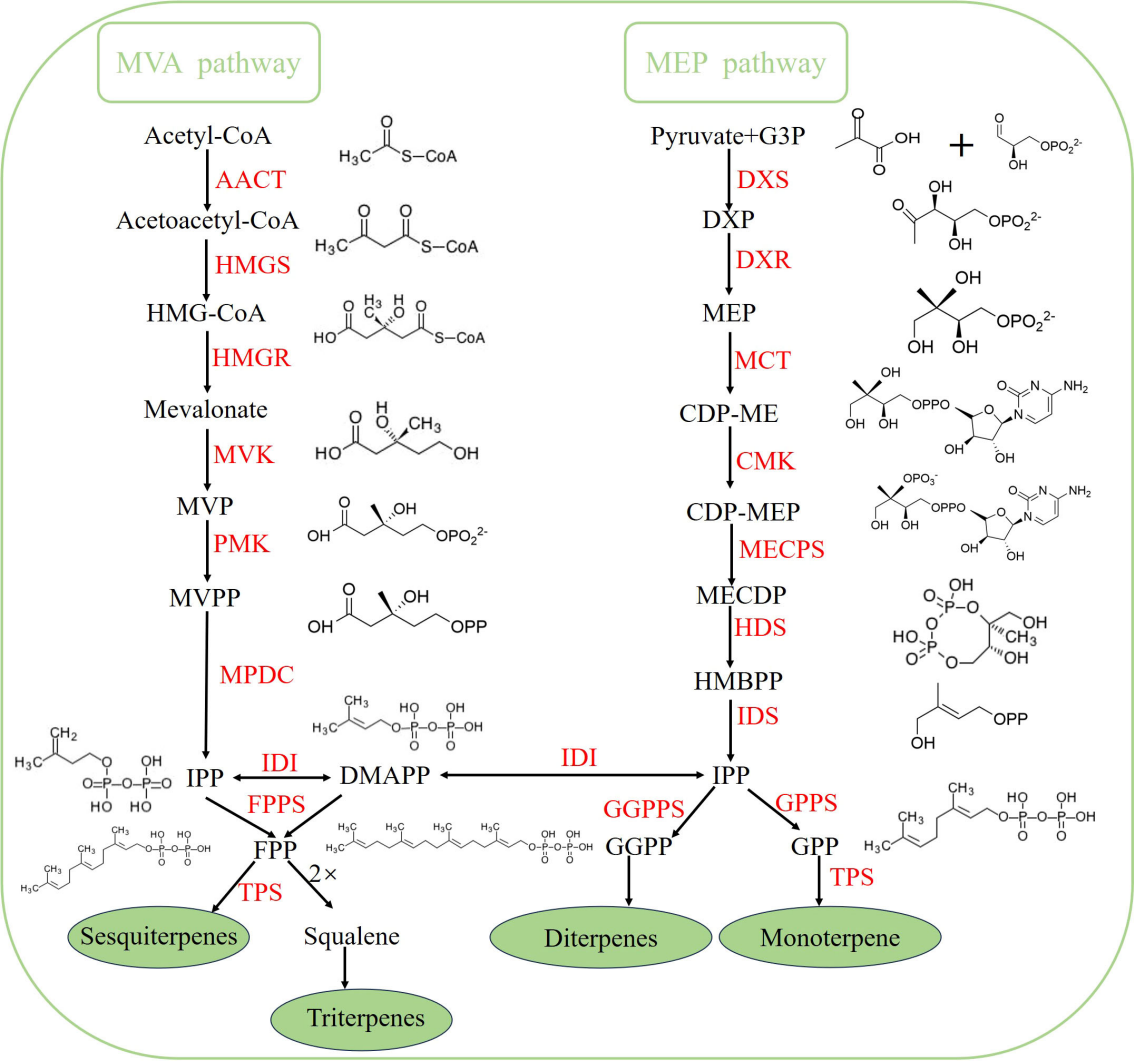
Terpenoid biosynthetic pathway. The key intermediates are shown with their chemical structures. # Acetyl-CoA, Acetyl coenzyme A; Acetoacetyl-CoA, Acetoacetyl coenzyme A; HMG-CoA, 3-hydroxy-3-methylglutaryl coenzyme A; MVP, Mevalonate 5-phosphate; MVPP, Mevalonate 5-diphosphate; G3P, D-glyceraldehyde 3-phosphate; DXP, 1-deoxy-D-xylulose 5-phosphate; MEP, 2-C-methyl-D-erythritol 4-phosphate; CDP-ME, 4-diphosphocytidyl-2-C-methyl-D-erythritol; CDP-MEP, 4-Diphosphocytidyl-2-C-methyl-D-erythritol 2-phosphate; MECDP, 2-C-methyl-D-erythritol 2, 4-cyclodiphosphate;HMBPP, (E)-4-hydroxy-3-methyl-but-2-enyl pyrophosphate; IPP, Isopentenyl diphosphate; DMAPP, Dimethylallyl diphosphate; GPP, Geranyl diphosphate; FPP, Farnesyl diphosphate; GGPP, Geranylgeranyl diphosphate; AACT; Acetyl-CoA C-acetyltransferase;HMGS, 3-Hydroxy-3-methylglutaryl-CoA synthase; HMGR, 3-Hydroxy-3-methylglutaryl-CoA reductase; MVK, Mevalonate kinase; PMK, Phosphomevalonate kinase; MPDC, Diphosphomevalonate decarboxylase;DXS, 1-Deoxy-D-xylulose 5-phosphate synthase;DXR, 1-Deoxy-D-xylulose 5-phosphate reductoisomerase; MCT, 2-C-methyl-D-erythritol 4-phosphate cytidylyltransferase; CMK, 4-Diphosphocytidyl-2-C-methyl-D-erythritol kinase; MECPS, 2-C-methyl-D-erythritol 2, 4-cyclodiphosphate synthase;HDS, 1-Hydroxy-2-methyl-2-(E)-butenyl 4-diphosphate synthase; IDS, Isopentenyl diphosphate synthase;IDI, Isopentenyl diphosphate delta-isomerase; FPPS, Farnesyl diphosphate synthase; GPPS, Geranyl diphosphate synthase; GGPPS, Geranylgeranyl diphosphate synthase;TPS, Terpene synthase.#.

Terpenoids are the most structurally diverse and widely distributed class of natural products, meaning they have the highest number of unique carbon skeletons and are found across the most species. They not only play a role in ecological processes such as plant defense, pollination attraction, and interspecific communication (Li et al., 2023), but also have a wide range of applications in the pharmaceutical, spice (Tetali, 2019), agricultural, and food industries (Fan et al., 2023). Research on terpenoid biosynthesis pathways is extensive, particularly in model plants. The key precursor substances for terpenoid biosynthesis are isopentenyl pyrophosphate (IPP) and its isomer dimethylallyl pyrophosphate (DMAPP) (Wang et al., 2019). These are mainly produced via two pathways: the mevalonic acid (MVA) pathway, which occurs in the cytoplasm, and the methylerythritol phosphate (MEP) pathway (Vranová et al., 2013), which takes place in plastids. Subsequently, linear precursors such as farnesyl pyrophosphate (FPP), geranyl pyrophosphate (GPP), and geranylgeranyl pyrophosphate (GGPP) are generated under the catalysis of enzymes. Under the action of terpene synthases, these linear precursors undergo complex reactions including cyclization and rearrangement to form structurally diverse terpene backbones, and finally, structurally diverse terpenoids are produced through a variety of modification reactions (**Figure 1**). Terpene synthases can be divided into multiple subfamilies such as TPSa-TPSh based on sequence similarity (Jia et al., 2022). Most sesquiterpenes belong to the TPS-a subfamily [(Hendrickson et al., 2024)], which has highly conserved structural characteristics, including the DDXXD motif composed of an aspartate-rich region (Chen et al., 2025). This motif is crucial for the binding of Mg^2+^ cofactors and the catalysis of substrates. In addition, some genes in the TPSb (Jones et al., 2011), TPS-g (Wang et al., 2024), and TPS-e/f (Huang et al., 2010)subfamilies can also synthesize sesquiterpenoids. However, the biosynthesis of sesquiterpenoids in *N. jatamansi* remains unclear. At the molecular level, only Feng et al (Feng et al., 2022) and Li et al (Li et al., 2025). have conducted relevant studies on it through transcriptomics.

To address the lack of clarity regarding genes associated with sesquiterpene synthesis in *N. jatamansi*, this study conducted research on the screening, cloning, and functional identification of sesquiterpene synthesis genes in this plant. First, we determined the volatile components in *N. jatamansi*. Combined with transcriptome data, we screened for sesquiterpene synthase genes in *N. jatamansi*. Bioinformatics and expression analyses were performed on these sesquiterpene synthase genes to select candidate genes involved in sesquiterpenoid synthesis. The functions of the candidate genes were further verified using the tobacco leaf transient expression system and yeast expression system. This study elucidates the molecular mechanism underlying the formation of the specific odor (a key quality trait) of *N. jatamansi*, laying a foundation for further ensuring the quality of *N. jatamansi* as a medicinal herb and promoting the sustainable development of its resources.

## 2 Results

### 2.1 Analysis of volatile components in *N. jatamansi*

A total of 736 metabolites were detected using the GC-MS platform and an in-house database (**ADDITIONAL FILE 1**). Terpenoids constituted the most abundant class with 211 compounds, accounting for 28.7% of the volatile components; Esters numbered 118, constituting 16% of the volatile components; heterocyclic compounds totaled 101, accounting for 13.7%; ketones comprised 60, representing 8.2%; hydrocarbons numbered 50, making up 6.8%; alcohols totaled 49, constituting 6.7%; 43 aromatic hydrocarbons, accounting for 5.8% of volatile components; 40 aldehydes, accounting for 5.4% of volatile components; 22 acids, accounting for 3.0% of volatile components; 13 phenols, accounting for 1.8% of volatile components; 11 amines, accounting for 1.5% of volatile components; 6 nitrogen-containing compounds, accounting for 0.8% of volatile components; 4 sulfur-containing compounds, accounting for 0.5% of volatile components; 3 halogenated hydrocarbon compounds, accounting for 0.4% of volatile components; 3 ether compounds, accounting for 0.4% of volatile components; and 2 other compounds, accounting for 0.3% of volatile components (**Supplementary Figure S1**).

### 2.2 Transcriptome sequencing and screening of candidate sesquiterpene synthase genes in *N. jatamansi*

The transcriptome sequencing analysis of *N. jatamansi* yielded a total of 14.29 Gb of clean data, with each sample achieving a clean data volume of 14 Gb. The Q30 base percentage was above 92%. A total of 80, 751 Transcript sequences were obtained, with an average sequence length of 1, 278 bp; 44, 944 Unigene sequences were obtained, with an average sequence length of 1, 592 bp. The Unigene sequences were aligned with the KEGG, Nr, Swiss-Prot, TrEMBL, KOG, GO, and Pfam databases, and the results are presented in **Supplementary Table S1**. Nine sesquiterpene synthase-related genes were identified from the genes annotated in the Nr database of the *N. jatamansi* transcriptome, named *NjTPS1* - *NjTPS9*.

### 2.3 Cloning of the *N. jatamansi* sesquiterpene synthase genes

Seven sesquiterpene synthase genes were successfully cloned via PCR amplification and vector ligation (**Supplementary Figure S2**), while no valid cloning products were obtained for *NjTPS3* and *NjTPS9*.

### 2.4 Bioinformatics analysis of candidate sesquiterpene synthase genes in *N. jatamansi*

**Figure 2:**
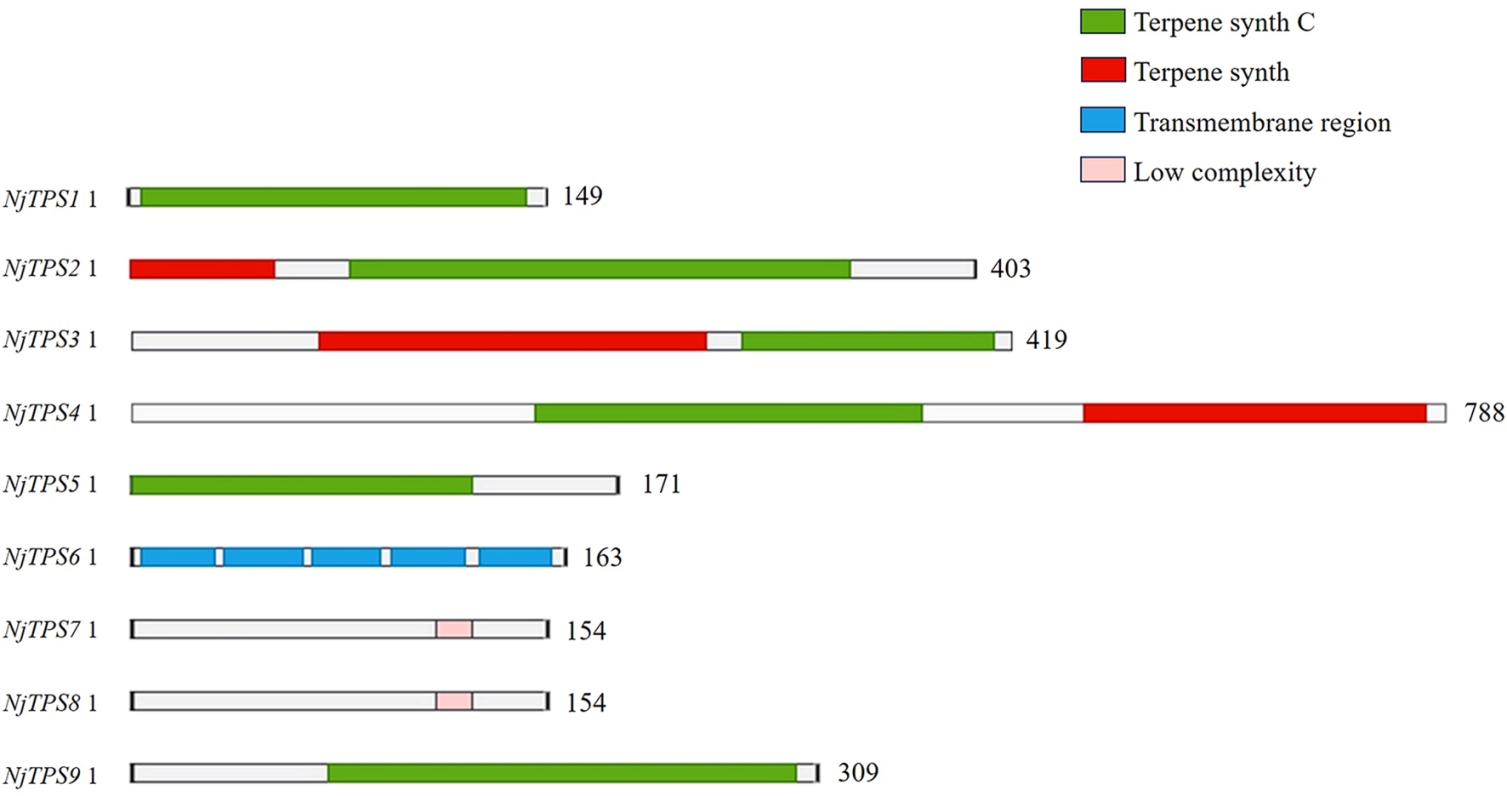
Domain analysis of sesquiterpene synthase from *N. jatamansi.* reprents Terpene synth C; reprents Terpene synth; represents transmembrane region; represents low complexity.

**Figure 3:**
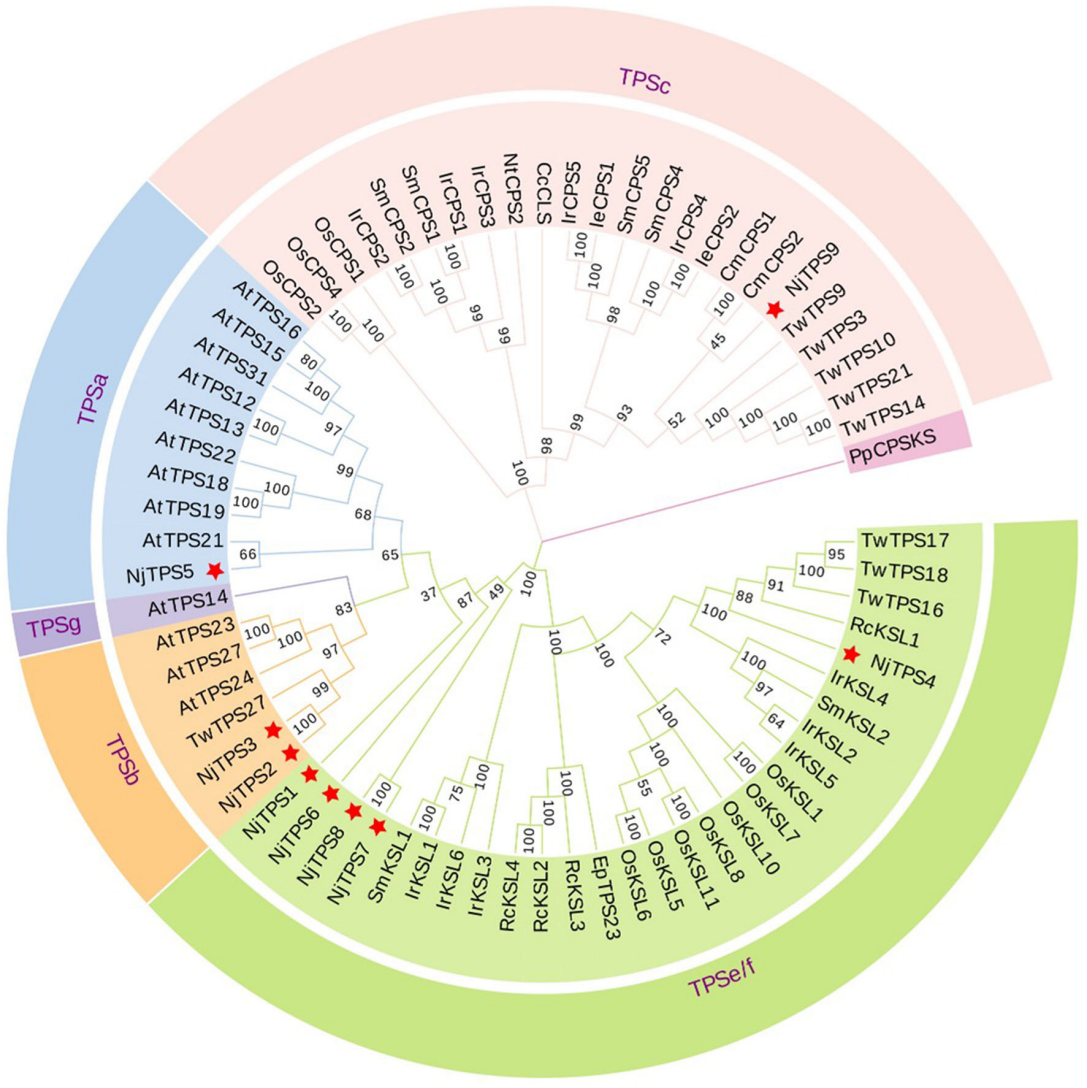
Phylogenetic analysis of candidate sesquiterpene synthases from *N. jatamansi*. Nj reprents *Nardostachys jatamansi*; At reprents *Arabidopsis thaliana*; Tw reprents *Tripterygium wilfordii*; Cm reprents *Cucurbita maxima*; Ie reprents *Isodon eriocalyx*; Ir reprents *Isodon rubescens*; Sm reprents *Salvia miltiorrhiza*; Nt reprents *Nicotiana tabacum*; Cc reprents *Cistus creticus*; Os reprents *Oryza sativa*; Rc reprents *Ricinus communis*; Ep reprents *Euphorbia peplus*. *Physcomitrella patens* copalyl diphosphate synthase/kaurene (*PpCPSKS*) was used as outgroup.

We analyze and predict the CDS length, physicochemical properties, and subcellular localization of candidate genes. The results are presented in **Supplementary Table S2**. Domain analysis (**Figure 2**) revealed that *NjTPS1*, *NjTPS4*, and *NjTPS9* contain the Terpene synthase C core catalytic domain, while the “Terpene synthase C” domain belongs to the C-terminal extended catalytic functional region, indicating that *NjTPS1*, *NjTPS4*, and *NjTPS9* may catalyze the production of a single product from the substrate. *NjTPS2*, *NjTPS3*, and *NjTPS4* contain both the Terpene synthase C and Terpene synthase core catalytic domains. The “Terpene synthase” domain is the core catalytic region basis of terpene synthases, containing conserved sequences required for the initiation of catalytic reactions, capable of binding to metal ions (such as Mg^2+^, Mn^2+^) and promoting the cyclization reaction of substrates (such as FPP), suggesting that *NjTPS2*, *NjTPS3*, and *NjTPS4* may catalyze the production of multiple complex products from the substrate. *NjTPS6* contains a transmembrane region domain, which is a transmembrane structure domain, indicating that *NjTPS6* may bind to membranes and affect subcellular localization or signal transduction. *NjTPS7* and *NjTPS8* contain low complexity domains, which are low-complexity regions composed of simple amino acid sequence repeats, suggesting that *NjTPS7* and *NjTPS8* may be involved in protein interactions, maintenance of flexible structures, etc. TPS family is divided into seven subfamilies, TPS-a to TPS-h, based on sequence similarity, functional characteristics and gene structure. TPS-c and TPS-e/f belong to the primary metabolic groups related to hormone synthesis. The former is mainly the synthesis of diterpenoid precursors catalyzed by bifunctional enzymes, while the latter is specifically involved in the production of gibberellin precursors, and the two are closely related; TPS-a and TPS-b mainly synthesize sesquiterpenes and monoterpenes, respectively. Some members have cross functions. TPS-g has unique versatility because it can simultaneously use monoterpenes and sesquiterpenes precursors; TPS-d is mainly distributed in gymnosperms and closely related to tps-h. it covers the synthesis of monoterpenes, sesquiterpenes and diterpenes, and is the core of defense substance synthesis in gymnosperms; TPS-h, a bifunctional diterpene synthase, only exists in Lycopodium plants (Jiang et al., 2019).The phylogenetic tree (**Figure 3**) shows that these *NjTPS* are classified into the TPS-a, TPS-b, TPS-c, and TPS-e/f subfamilies.

### 2.5 Expression analysis of candidate sesquiterpene synthase genes from *N. jatamansi*

**Figure 4:**
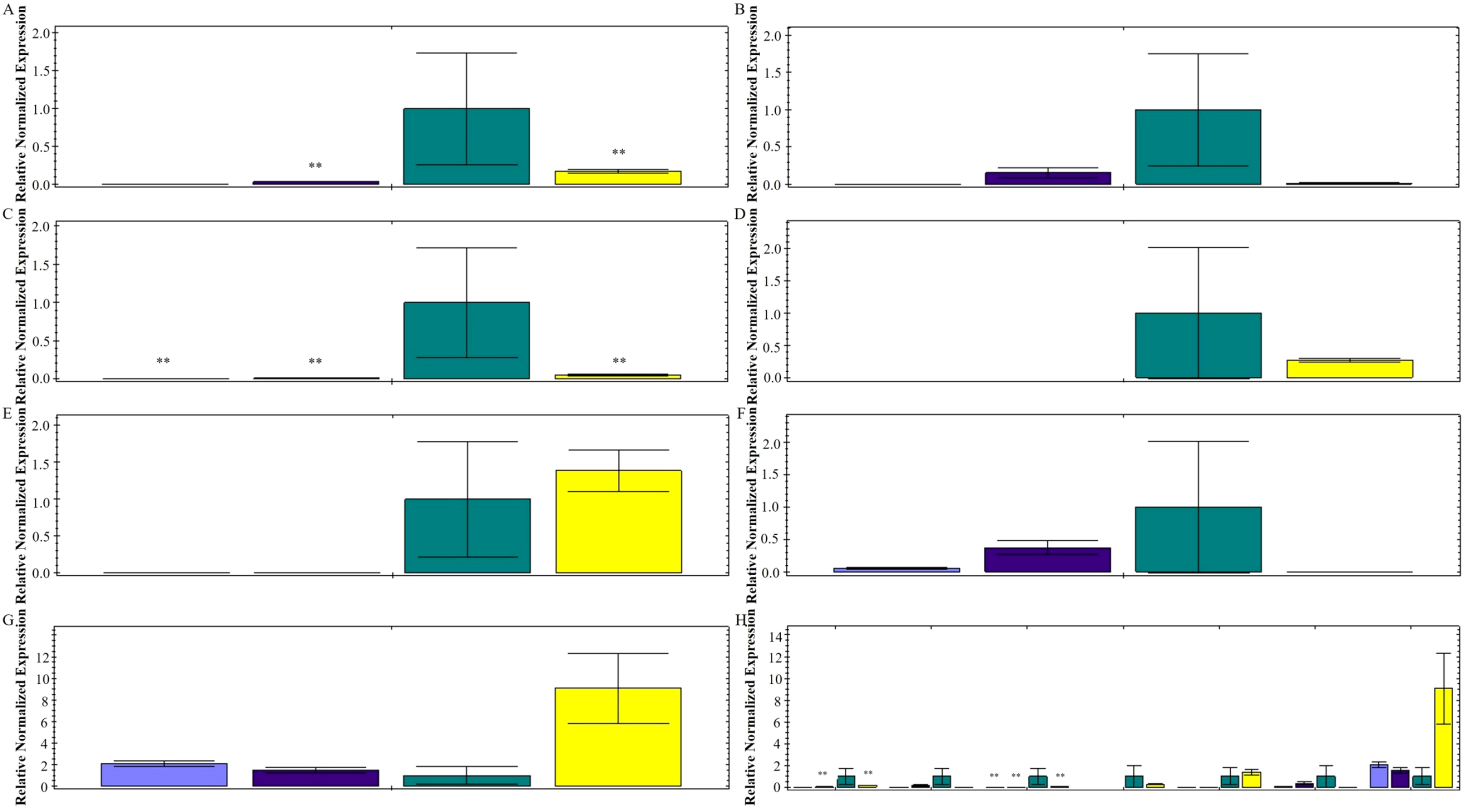
Expression analysis of sesquiterpene synthase genes in different tissues of *N. jatamansi*. Figures **(A–G)** represent the relative expression levels of genes *NjTPS1*, *NjTPS2*, *NjTPS5*, *NjTPS6*, *NjTPS7*, and *NjTPS8* in different tissues, respectively; Figure **(H)** shows the relative expression levels of the 7 genes in the four tissues. Green represents roots, yellow represents stems, dark purple represents leaves, and light purple represents flowers. **P<0.01.

RNA was extracted from four tissues of *N. jatamansi* (roots, stems, leaves, and flowers) respectively, and then reverse-transcribed into cDNA. Using the cDNA as a template, qRT-PCR was performed to determine the expression levels of the 7 successfully cloned candidate sesquiterpene synthase genes from *N. jatamansi*. The results (**Figure 4**) showed that *NjTPS1*, *NjTPS2*, *NjTPS4*, and *NjTPS7* all had the highest expression levels in roots, with relatively lower expression levels in other tissues; *NjTPS5* had the highest expression level in roots but was not expressed in leaves and flowers; *NjTPS6* and *NjTPS8* had the highest expression levels in stems, with lower expression levels in other tissues. Among these 7 genes, *NjTPS5* exhibited the highest expression level in roots, while *NjTPS8* had the lowest; in stems, *NjTPS8* showed the highest expression level, and *NjTPS7* had the lowest; in leaves, *NjTPS8* had the highest expression level, while *NjTPS6* was the lowest; in flowers, *NjTPS8* displayed the highest expression level, and *NjTPS1* had the lowest.

### 2.6 *In vivo* functional verification of *NjTPS2*

Through domain analysis and phylogenetic analysis, it was found that *NjTPS6*, *NjTPS7*, and *NjTPS8* do not contain core domains for terpenoid synthesis; *NjTPS1* and *NjTPS4* belong to the TPSe/f subfamily and generally do not catalyze the formation of sesquiterpenes from FPP; After experimental analysis, it was found that *NjTPS5* had low activity, so *NjTPS2* was ultimately chosen for subsequent functional validation experiments.

**Figure 5:**
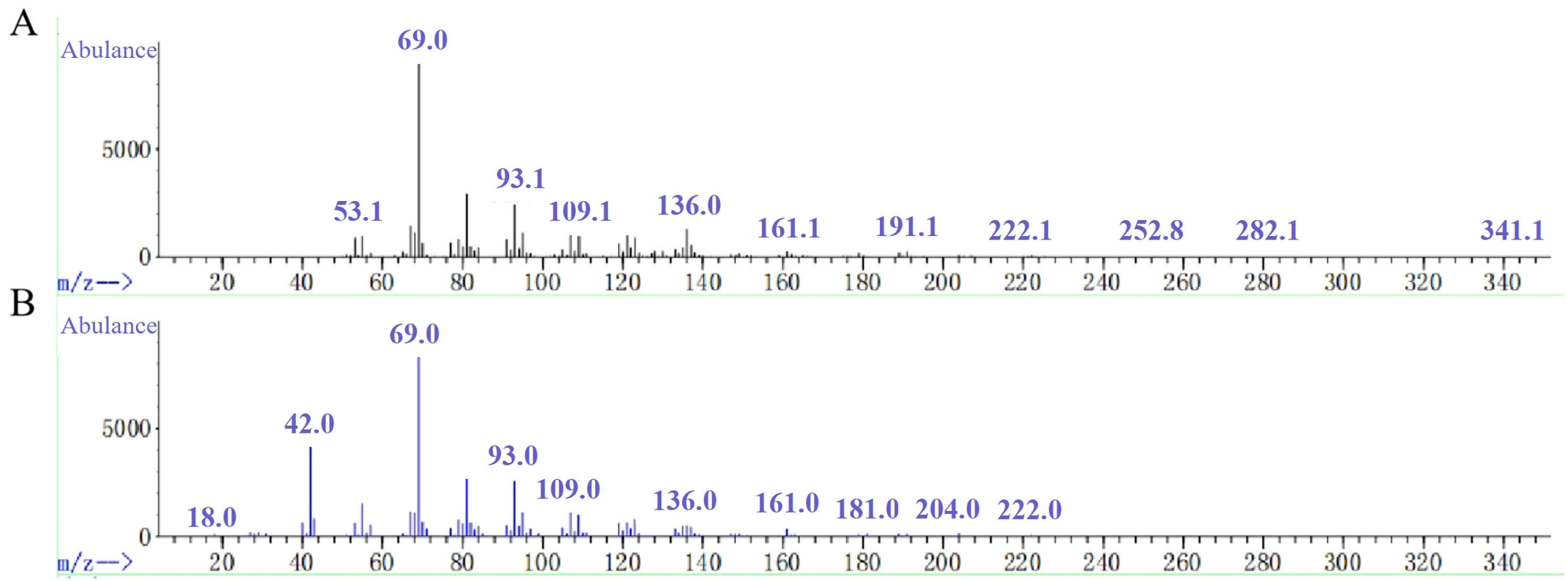
GC-MS detection results of the heterologous expression products of the *NjTPS2* gene in yeast. **(A)** is the mass spectrum of the tested compound, and **(B)** is the mass spectrum of farnesol in the database. When detected under the same experimental conditions, the target compound was not detected in the blank group.

The empty *pDEST* vector and the recombinant *pDEST-NjTPS2* vector were respectively transformed into yeast engineering strains containing FPP. The components were detected by GC-MS, and the results were compared in the database. The component determination results showed that compared with the empty *pDEST* vector, *NjTPS2* produced two terpenoid compounds in yeast (**Figure 5**). The two terpenoid compounds were farnesol, a sesquiterpenoid, at 9.116 minutes.

**Figure 6:**
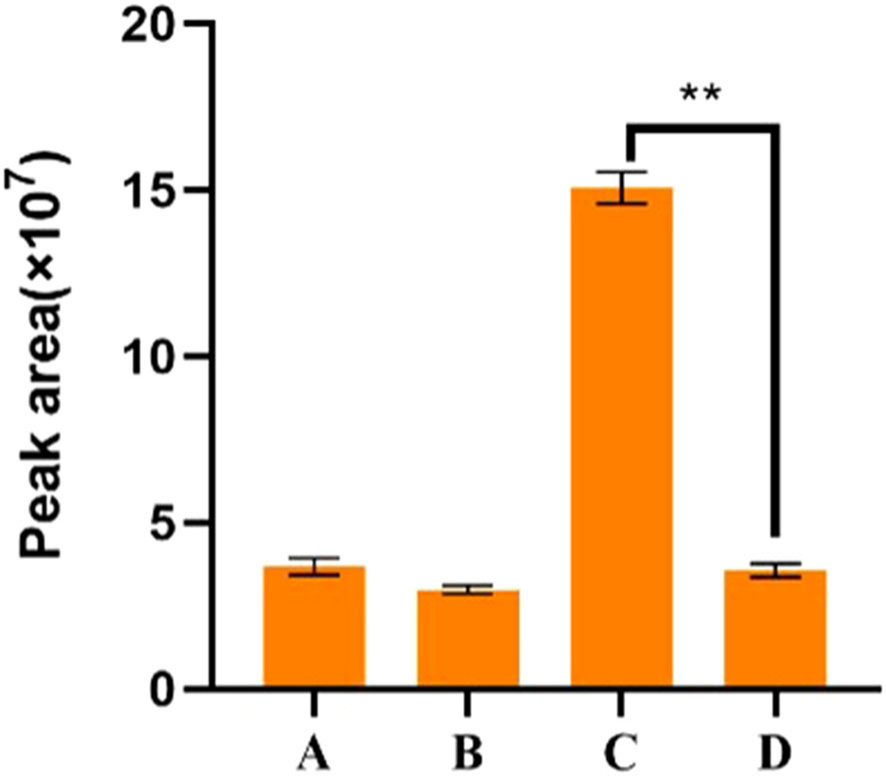
LC-MS detection results of *NjTPS2* expression in tobacco. **(A)** Peak area of α-cyperone in the NjTPS2 heterologous expression experimental group; **(B)** Peak area of 2-[(1S, 2S, 4aR, 8aS)-1-hydroxy-4a-methyl-8-methylene-1, 2, 3, 4, 5, 6, 7, 8a-octahydronaphthalen-2-yl]prop-2-enoic acid in the NjTPS2 heterologous expression experimental group; **(C)** Peak area of NP-004917 in the NjTPS2 heterologous expression experimental group; **(D)** Peak area of NP-004917 in the blank control group without NjTPS2 expression. Data are presented as mean ± SE, and statistical analysis was performed using independent samples t-test. **P<0.01 indicates an extremely significant difference between C and D.

The empty *pEAQ* vector and the recombinant *pEAQ-NjTPS2* vector were respectively transformed into Agrobacterium, which was then used to infiltrate tobacco. High-resolution mass spectrometry was employed for detection, and the results were compared against a database. The component determination results showed that, compared with tobacco infiltrated with the empty *pEAQ* vector, the tobacco infiltrated with the recombinant *pEAQ-NjTPS2* vector produced two new sesquiterpenoid compounds (**Figure 6**), namely α-cyperone and 2-[(1S, 2S, 4aR, 8aS)-1-hydroxy-4a-methyl-8-methylene-1, 2, 3, 4, 5, 6, 7, 8a-octahydronaphthalen-2-yl]prop-2-enoic acid. Both groups of tobacco contained an unknown sesquiterpenoid compound, NP-004917. Calculations revealed that the peak area of this compound in 100 mg of tobacco leaves harboring the empty *pEAQ* vector was 3.5×10^7^, while its peak area in 100 mg of tobacco leaves harboring the *pEAQ-NjTPS2* vector was 1.56×10^8^. Therefore, it was inferred that *NjTPS2* can promote the production of NP-004917.

## 3 Discussion

*N. jatamansi* is a commonly used and endangered Sino-Tibetan medicinal herb with clear efficacy, extensive clinical application, and high market demand. However, previous studies have mostly focused on its pharmacological effects and chemical composition, while production-related research has primarily centered on its cultivation. At the molecular level, investigations have been limited to transcriptomic analyses. Feng et al (Feng et al., 2022). Revealed the framework for the accumulation and biosynthesis of sesquiterpenoids in plant tissues through transcriptomic and metabolomic analyses; however, studies on the biosynthetic mechanism of sesquiterpenoids in *N. jatamansi* and their key genes remain scarce. Li et al (Li et al., 2025). identified a gene cluster potentially involved in sesquiterpene synthesis in *N. jatamansi* via transcriptomic analysis, but functional verification of the screened genes was not conducted.

Based on the GC-MS analysis of *N. jatamansi*, we detected a total of 736 compounds, which were classified into 16 categories. Among these, terpenoid compounds were the most abundant, with 211 species accounting for 28.7% of the volatile components; there were 138 sesquiterpenoid compounds, accounting for 65.4% of the terpenoid compounds. Feng et al. detected the volatile components of *N. jatamansi* via GC-MS and successfully identified a total of 65 compounds, including 50 sesquiterpenoid compounds (Feng et al., 2022). Although modern research has confirmed that the TPS-a subfamily is the most major and concentrated subfamily of sesquiterpene synthases, in some plants, certain TPS-b members also “moonlight” with the ability to utilize FPP for sesquiterpene synthesis. The vast majority of genes responsible for sesquiterpene synthesis share a common ancestor, which may initially have had relatively broad or simple functions (Liang et al., 2021). Throughout the long evolutionary history of plants, this ancestral gene has undergone multiple gene duplication events. These duplicated genes have evolved new functions enabling them to catalyze different substrates (FPP or its isomers) and generate a variety of sesquiterpene backbones—a process known as lineage-specific expansion. Despite sequence divergence, they all retain the conserved structural motifs common to terpene synthases, such as the DDXXD motif associated with substrate and magnesium ion binding (Ee et al., 2014), which serves as evidence of their homology.

Most existing studies on sesquiterpene synthases in *N. jatamansi* have focused on transcriptome analysis, expression analysis, and hormone treatment. Tang et al. successfully screened out candidate sesquiterpene synthase genes of *N. jatamansi* (including *NjTPS-49*, *NjTPS-54*, *NjTPS-56*, *NjTPS-57*, and *NjTPS-59* by performing transcriptome sequencing and treating the candidate genes of sesquiterpene synthases with MeJA (Tang et al., 2024). Feng et al. revealed the framework for the accumulation and biosynthesis of sesquiterpenoids in plant tissues through transcriptomic and metabolomic analyses; however, there is limited research on the biosynthetic mechanism of sesquiterpenoids in *N. jatamansi* and their key genes (Feng et al., 2022). Li et al. identified a gene cluster potentially involved in sesquiterpene synthesis in *N. jatamansi* by analyzing the plant’s transcriptome (Li et al., 2025). Nevertheless, none of these studies have conducted functional identification of the candidate sesquiterpene synthase genes. In our research, functional characterization of the *NjTPS2* gene was conducted using tobacco and yeast systems. Expression of the *NjTPS2* gene in yeast revealed that it can produce a sesquiterpenoid compound, trans-farnesol. In contrast, when *NjTPS2* was expressed in tobacco, it generated two sesquiterpenoid compounds—α-cyperone and 2-[(1S, 2S, 4aR, 8aS)-1-hydroxy-4a-methyl-8-methylene-1, 2, 3, 4, 5, 6, 7, 8a-octahydronaphthalen-2-yl]prop-2-enoic acid—and also promoted the production of the sesquiterpenoid compound NP-004917.

**Figure 7:**
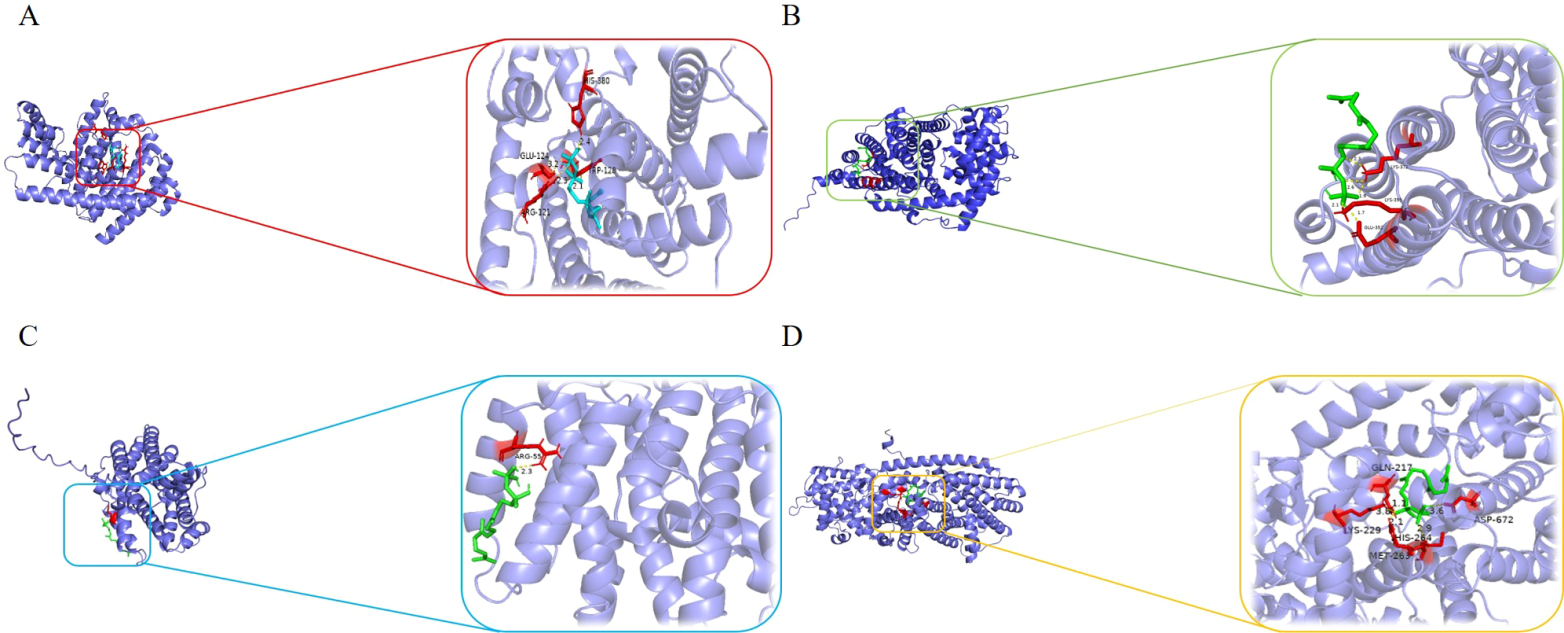
Molecular docking simulation results of different genes with FPP respectively. **(A)** represents the docking result of *NjTPS2* with FPP; **(B)** represents the docking result of *AsHS1* with FPP; **(C)** represents the docking result of *GS11330* with FPP; **(D)** represents the docking result of *STPS3* with FPP. Blue represents the three-dimensional structure of each protein; green represents the molecular structure of the substrate FPP; red represents the key binding residues involved in the interaction between the protein and the substrate FPP.

At the same time, we also compared the docking results of *NjTPS2* and FPP with the reported sesquiterpene synthase genes of other plants by molecular docking, and analyzed the mechanism of *NjTPS2* and FPP. The protein structures of *NjTPS2*, *ASHS1*, *GS11330* and *STPS3* were predicted by using the online website Alpha Fold 2. The FPP structure was downloaded from the online website PubChem. The molecular docking of *NjTPS2*, *ASHS1*, *GS11330* and *STPS3* with FPP was performed by using autodockvina. (**Figure 7**). The docking results showed that *NjTPS2* and FPP are connected via hydrogen bonds at residues GLU-124, ARG-121, TRP-128, and HIS-380, respectively. And the docking binding energy is – 7.3 kcal/mol. For the previously reported sesquiterpene synthases from other species: *AsHS1* from *Aquilaria sinensis* forms hydrogen bonds with FPP at residues GLU-352, LYS-355, and LYS-372 (Ran et al., 2023); *GS11330* (a reported sesquiterpene synthase from *Ganoderma sinense*) interacts with FPP via a hydrogen bond at residue ARG-55 (Cao et al., 2022); and *STPS3*, a sesquiterpene synthase from *Setaria italica* connects with FPP through hydrogen bonds at residues GLN-217, LYS-229, MET-263, HIS-264, and ASP-672 (Karunanithi et al., 2020). The above results indicate that *NjTPS2* can interact with FPP to produce sesquiterpenoid compounds.

The experimental results showed that the NjTPS2 gene exhibited distinct product profiles in the two heterologous expression systems of yeast and tobacco. To explore the universality of this phenomenon, we conducted a literature review and found that it is quite common for the same terpene synthase gene to yield different product spectra across various heterologous expression systems.For example, researchers such as Kanagarajan (Kanagarajan et al., 2012) cloned and functionally characterized amorpha-4, 11-diene synthase (ADS) derived from Artemisia annua in a tobacco transient expression system. Their results demonstrated that ADS predominantly produced amorpha-4, 11-diene in tobacco leaves, along with only one minor by-product. In contrast, previous studies confirmed that the same enzyme generated at least 15 trace by-products in the Escherichia coli prokaryotic expression system. This finding verified the significant impact of heterologous expression systems on the product spectra of terpene synthases.Combined with literature analysis and existing research, we propose that the core reason for the differential product formation of the same gene in different expression systems may lie in the inherent differences in host metabolic backgrounds and functional networks (Grabherr et al., 2011). Yeast lacks plant-specific cofactors dedicated to terpene synthesis as well as endogenous modification enzyme systems, resulting in a relatively simple composition of terpene precursor pools and metabolic flux directions. In contrast, as a higher plant, tobacco possesses a complete terpene synthesis network. The endogenous metabolic enzymes in tobacco cells can further transform and modify the synthesized products, and also perform plant-specific post-translational modifications (e.g., glycosylation and phosphorylation) on terpene synthases.

In conclusion, through RNA-seq sequencing of *N. jatamansi*, we successfully screened out 9 candidate sesquiterpene synthase genes. Cloning and bioinformatics analysis of *NjTPS2* revealed that it contains two core terpene catalytic domains, namely Terpene synth C and Terpene synth. The results of quantitative real-time PCR (qRT-PCR) showed that *NjTPS2* had the highest expression level in roots and relatively low expression levels in other tissues. Functional verification of *NjTPS2* was conducted using the tobacco transient expression system and yeast expression system, and it was found that *NjTPS2* could produce sesquiterpenoid, confirming that *NjTPS2* has the functional activity of a sesquiterpene synthase. This study provides insights into the molecular mechanism underlying the quality formation of *N. jatamansi* and offers genetic resources for the biosynthesis of sesquiterpenoids in *N. jatamansi* and the breeding of high-quality *N. jatamansi* varieties.

## 4 Materials and methods

### 4.1 Plant materials

The *N. jatamansi* sample used in the study was collected from the Sichuan Academy of Grassland Sciences in August 2022, and its fresh rhizomes were used for transcriptome sequencing. Four tissues, including roots, stems, leaves, and flowers, of the fresh *N. jatamansi* sample were frozen at -80°C for subsequent experiments.

### 4.2 Determination of volatile components in *N. jatamansi*

After liquid nitrogen grinding and vortex mixing, the root and rhizome samples of *N. jatamansi* were weighed and placed in a headspace bottle, saturated NaCl solution and 10 μL (50 μg/ml) internal standard solution were added, and the 120 µm dvb/cwr/pdms extraction head was treated by fully automatic headspace solid phase microextraction (HS-SPME) (head space extraction for 15 min after shaking at 60°C for 5 min, desorption at 250°C for 5 min) for GC-MS analysis; The instrument model is Agilent 8890-7000D. The GC was performed on a db-5ms capillary column (30 m × 0.25 mm × 0.25 μm, Agilent j&w scientific) with high-purity helium (≥ 99.999%) as the carrier gas (constant flow 1.2 mL/min). The injection port was 250°C, the sample was injected without partial flow, and the solvent was delayed for 3.5 min. the programmed temperature was maintained at 40°C for 3.5 min, and then increased to 100°C, 180°C, and 280°C for 5 min at 10°C, 7°C, and 25°C/min, respectively; MS uses the electron bombardment ion source (EI, electron energy 70ev), and the interface temperatures of ion source, quadrupole and mass spectrometry are 230°C, 150°C and 280°C, respectively. The qualitative and quantitative ions are accurately scanned in the selected ion detection (SIM) mode. An in-house database was established based on multi-species references, published literature, partial standard substances and retention indices, which contains the confirmed retention time (RT), qualitative ions and quantitative ions for SIM analysis.For each compound, one quantitative ion and 2–3 qualitative ions were selected. The target ions in each group were monitored in different time windows according to their peak elution order. A compound was identified if its detected retention time matched the standard reference value, and all the preset target ions were present in the sample mass spectrum after background subtraction.The quantitative ions were selected for peak integration and calibration to improve the accuracy of quantification.

### 4.3 RNA extraction and transcriptome sequencing

Following the manufacturer’s instructions, total RNA was extracted from *N. jatamansi* root and rhizome using an RNA extraction kit supplied by TransGen Biotech (China). RNA integrity and potential DNA contamination were evaluated through 1.5% agarose gel electrophoresis. RNA purity was measured using a NanoPhotometer spectrophotometer, while RNA concentration was precisely quantified with a Qubit 2.0 fluorometer (Life Technologies, Carlsbad, CA, USA). Additionally, RNA integrity was accurately assessed using the Agilent 2100 Bioanalyzer. Library construction and transcriptome sequencing were performed by Metware Biotechnology Co., Ltd. on the Illumina sequencing platform, using a pooled RNA sample extracted from the roots and rhizomes of three *N. jatamansi* plants with consistent growth status as the template. The original sequencing data were filtered to obtain high-quality reads, and then spliced to obtain transcriptome. We used the fastp software (Chen et al., 2018) to perform stringent quality control on the sequencing data for the purpose of obtaining high-quality sequencing data. The specific filtering criteria are listed as follows:Removal of sequencing reads with adapter sequences attached;Exclusion of paired-end reads if the proportion of N bases in either read exceeds 10% of the total base number of that read;Exclusion of paired reads if the proportion of low-quality bases (Q ≤ 20) in either read exceeds 50% of the total base number of that read. After obtaining clean reads, Trinity (Grabherr et al., 2011) was used to splice clean reads to obtain reference sequences for subsequent analysis. Trinity software is described as follows:A k-mer dictionary (k = 25) was constructed from the raw sequences, and seed k-mers were selected and extended bidirectionally to generate contigs. A set of contigs was obtained accordingly and then subjected to clustering. After clustering, a *de novo* de Bruijn graph was reconstructed for each cluster to facilitate subsequent assembly. The generated graphs were trimmed to remove minor edge paths, with only the major paths retained. Finally, the Butterfly module aligned the raw sequences against these major paths, and the paths with substantial support from the raw sequences were preserved to yield the final assembly results. The transcripts were hierarchical clustered by using the reads number and expression pattern of transcripts on corset alignment. The transcripts obtained by Trinity splicing were used as reference sequences for subsequent analysis. The longest cluster sequence obtained after corset hierarchical clustering is used as UniGene for subsequent analysis.

### 4.4 Synthesis of cDNA and cloning of candidate sesquiterpene synthase genes

A reverse transcription kit (TaKaRa, China) was employed to reverse transcribe equal amounts of mixed RNA from the roots and rhizomes of *N. jatamansi* into cDNA, following the manufacturer’s instructions. Using the mixed cDNA as a template, specific primers were utilized to amplify the candidate genes. Subsequently, the amplified candidate genes were analyzed via 1.5% agarose gel electrophoresis to verify the accuracy of the candidate gene band sizes. The gene bands of the correct size were then recovered using a DNA purification and recovery kit (Tiangen, China). The primer sequence is shown in **Supplementary Table S3**. The recovered DNA fragment was used as a template and ligated with the pMD™19-T vector (TaKaRa, China) following the instructions. The alignment of candidate genes was conducted using DNAMAN software.

### 4.5 Bioinformatics analysis of candidate sesquiterpene synthase genes

Utilize the online tool ORFfinder to predict the open reading frame and amino acid sequence of the sequence, and employ software such as ProtParam, Cell-PLoc 2.0, Pfam, and SMART to predict the physicochemical properties, subcellular localization, and structural domains of the candidate gene. The candidate gene for *N. jatamansi* sesquiterpene synthase, along with the TPS gene from *Arabidopsis thaliana* and previously reported terpene synthase genes from other species, was utilized to construct a phylogenetic tree using MEGA12, and the phylogenetic tree was constructed via the Neighbor-Joining (NJ) method, with the reliability of the branches evaluated by a bootstrap test with 3000 replicates. Physcomitrella patens copalyl diphosphate synthase/kaurene (*PpCPSKS*) was used as outgroup (Mao et al., 2021). Subsequently, gene subfamily analysis was performed.

### 4.6 Expression analysis of candidate sesquiterpene synthase genes

For Real-time quantitative PCR analysis, RNA was first extracted from the roots, stems, leaves, and flowers of *N. jatamansi* using an RNA extraction kit. Then, the RNA was reverse transcribed into cDNA using an RNA reverse transcription kit. Real-time quantitative polymerase chain reaction was performed using the SYBR Green reagent kit (TaKaRa, China). The primer sequences are provided in **Supplementary Table S4**. The results were normalized using the internal reference gene GAPDH. Relative expression levels were calculated as the average of three technical replicates. Using Duncan’s multiple range test (P<0.01) in SPSS software to analyze expression differences in different tissues.

### 4.7 Transformation of yeast engineering strain and component determination

Using the Frozen-EZ Yeast Transformation II Kit (Coolaber, China), yeast competent cells were prepared according to the instructions. The *pDEST-NjTPS2* recombinant expression vector was constructed using Gateway™ LR Clonase™ II (Invitrogen, US). Take 0.5 μg of *pDEST-NjTPS2* recombinant plasmid and add it to 100 μL of yeast competent cells. Mix gently and incubate. After incubation, centrifuge at 2000×g for 2 minutes at room temperature, discard the supernatant, and retain 200 μL of the yeast solution. Pipette and mix the solution, then spread it onto a synthetic auxotrophic SD-Ura plate and incubate it upside down at 30°C for 72 hours. Randomly pick a single colony from the SD-Ura plate and inoculate it into 2mL of SD-Ura liquid medium containing 2% glucose. Incubate the mixture at 30°C and 200rpm for 24 hours. Pipette 10 μL of yeast solution into a PCR tube, add 10 μL of lysis buffer, and incubate at 99°C for 20 minutes to lyse the yeast cells. Take 2 μL of the bottom layer of lysis fragment yeast solution for positive clone PCR, and verify by 1.5% agarose gel electrophoresis whether the plasmid has been successfully transformed into Saccharomyces cerevisiae. Use the verified yeast solution for fermentation. Take 100 μL of PCR-positiveyeast solution and inoculate it into 5 mL of YPD medium, and incubate overnight at 30°C and 200 rpm. Take 1mL of yeast solution and transfer it into a sterile shake flask containing 50 mL of YPD medium, and incubate at 30°C and 200rpm for 72 hours for fermentation. Collect the fermented yeast solution, centrifuge it, discard the supernatant, add 2mL of chromatographic-grade ethyl acetate to the yeast cells, subject to ultrasonic extraction at low temperature for 30 minutes, repeat the extraction once, centrifuge the extract at high speed, collect the upper ethyl acetate phase, and detect it using GC-MS. Automatic injection was performed with an injection volume of 1 μL in split mode (split ratio = 20:1). The initial temperature was held at 60°C for 1.5 min, then increased to 200°C at a rate of 25°C/min, followed by a further increase to 300°C at a rate of 5°C/min, and maintained at this temperature for 10 min. The injector temperature was set at 250°C, the ion source temperature was 250°C, and the electron energy was 90 eV. A mass-to-charge ratio (m/z) range of 50–1000 was scanned for all detected samples.

### 4.8 Tobacco transformation and component determination

Utilize Gateway™ LR Clonase™ II to construct the *pEAQ*-*NjTPS2* recombinant expression vector. Transfer the *pEAQ* empty vector and the *pEAQ-NjTPS2* recombinant vector into Agrobacterium tumefaciens GV3101. Take 100 μL of the transformed bacterial solution and spread it onto a LB agar plate containing 50 μg mL^-1^ kanamycin and 20 μg mL^-1^ rifampin. Culture at 28°C for 3 days. Pick a single bacterial colony and inoculate it into 1mL of the same resistant LB liquid medium. Incubate the culture at 28°C with shaking at 200 rpm for 24 hours. Transfer the activated bacterial solution to 50 mL of the same resistant LB liquid medium and incubate it at 28°C with shaking at 200 rpm until the OD600 reaches 1.0. Centrifuge the culture at 6000 rpm at room temperature (approximately 25°C), discard the LB medium, and collect the bacterial cells. Prepare MMA infection solution (containing 200 μM acetylsyringone, 10 mM MgCl_2_, and 10 mM MES), adjust the pH to 5.6. Resuspend the bacterial cells in the MMA infection solution to an OD600 of 1.0, and incubate at 28°C with shaking at 50 rpm for 2 hours. Select tobacco plants grown in a greenhouse for 4 to 6 weeks, use a disposable syringe without a needle to extract the aforementioned empty vector and recombinant vector bacterial solution, inject it into the tobacco leaves, and culture for 5 days. Freeze-dry the infected leaves and grind them into powder. Weigh 70mg of empty vector tobacco leaf powder and 100mg of recombinant vector tobacco leaf powder, add 1mL of methanol to each, vortex for 5 minutes, and then ultrasonicate at 60°C for 30 minutes. The mixture is centrifuged at 5000 rpm for 15 minutes at room temperature (approximately 25°C), and the supernatant is collected. High-resolution mass spectrometry is used to analyze the supernatant.

## Data Availability

The datasets presented in this study can be found in online repositories. The names of the repository/repositories and accession number(s) can be found below: https://www.ncbi.nlm.nih.gov/, PRJNA1335842.

## Supplementary material

ADDITIONAL FILE 1 This file is a table containing the volatile metabolites of *N. jatamansi*

ADDITIONAL FILE 2 This file includes all additional tables (**Supplementary Tables S1**-**S4**) used in this manuscript. Table numbers and titles were listed as follows:
