# Supplementary material for: Cloning and functional identification of sesquiterpene synthase gene NjTPS2 in Nardostachys jatamansi DC
Source: Front Plant Sci. 2026 Jan 12;16:1718102. doi: 10.3389/fpls.2025.1718102 (PMC12833414; doi:10.3389/fpls.2025.1718102)
Supplement: ADDITIONAL FILE 2 — This file includes all additional tables (Supplementary Tables S1-S4) used in this manuscript. Table numbers and titles were listed as follows: [file DataSheet1.docx]

Supplementary Table 1: Statistical Table of Annotations

| Database | Number of Genes | Percentage (%) |
| --- | --- | --- |
| KEGG | 27217 | 60.56 |
| Nr | 35124 | 78.15 |
| SwissProt | 26742 | 59.50 |
| TrEMBL | 35096 | 78.09 |
| KOG | 21864 | 48.65 |
| GO | 30631 | 68.15 |
| Pfam | 27574 | 61.35 |
| Annotated in at least one Database | 35560 | 79.12 |
| Total Unigenes | 44944 | 100.00 |

Supplementary Table 2: Bioinformatics Analysis of Candidate Sesquiterpene Synthases

| Protein | CDS Length/bp | Number of Amino Acids | Molecular Formula | Molecular Weight/kDa | Isoelectric Point | Acidity and Alkalinity | Hydrophobicity | Subcellular Localization | Nr Database |
| --- | --- | --- | --- | --- | --- | --- | --- | --- | --- |
| NjTPS1 | 450 | 149 | C_794_H_1221_N_197_O_224_S_4_ | 17.24 | 5.43 | Acidity | 0.073 | Cytoplasm/Nucleus | hypothetical protein |
| NjTPS2 | 1212 | 403 | C_2090_H_3261_N_539_O_618_S_23_ | 46.56 | 4.79 | Acidity | -0.198 | Cytoplasm | geraniol synthase |
| NjTPS3 | 1260 | 419 | C_2151_H_3433_N_573_O_643_S_16_ | 48.12 | 6.24 | Acidity | -0.234 | Cytoplasm/Nucleus | sesquiterpene synthase 3 |
| NjTPS4 | 2412 | 788 | C_4019_H_6301_N_1069_O_1205_S_42_ | 90.22 | 5.55 | Acidity | -0.289 | Cytoplasm | hypothetical protein |
| NjTPS5 | 534 | 171 | C902H_1396_N_230_O_266_S_6_ | 19.91 | 4.97 | Acidity | -0.228 | Cytoplasm | sesquiterpene synthase 3 |
| NjTPS6 | 489 | 163 | C_930_H_1459_N_213_O_196_S_12_ | 19.14 | 9.99 | Alkalinity | 1.394 | Cytoplasmic Membrane | gag-pol polyprotein |
| NjTPS7 | 462 | 154 | C_749_H_1154_N_222_O_242_S_6_ | 17.33 | 5.8 | Acidity | -0.862 | Extracellular | hypothetical protein |
| NjTPS8 | 462 | 154 | C_749_H_1154_N_222_O_242_S_6_ | 17.33 | 5.8 | Acidity | -0.862 | Extracellular | hypothetical protein |
| NjTPS9 | 927 | 309 | C_1620_H_2505_N_445_O_472_S_17_ | 36.31 | 8.11 | Alkalinity | -0.504 | Extracellular/Nucleus | sesquiterpene synthase 3 |

Supplementary Table 3 Cloning Primers for Candidate Sesquiterpene Synthase Genes

| Gene | Primer |
| --- | --- |
| NjTPS1 | F:ATGGGTGAAAAAAGTAGAATGA |
|  | R:TTAATTAGCTCCTCGATCTTTA |
| NjTPS2 | F:ATGGGAATGTTGAGCTTGTATG |
|  | R:TTACATACGAATTGGTGTATAA |
| NjTPS3 | F:ATGATATCCTCATCATCGGTAC |
|  | R:TTATTTGAAATACAAATATGTT |
| NjTPS4 | F:AGGTGCTTGATCTCTTTTGACA |
|  | R:TTATTCGAGTTGGTGTTCTTCA |
| NjTPS5 | F:ATCGTTAGGTGCTACGATATGG |
|  | R:TTATATGGATACGGGATCTACG |
| NjTPS6 | F:ATGCAAAATATTCCCCTTCTGA |
|  | R:TGTCACAATACATAGGAGTAGG |
| NjTPS7 | F:ATGGCGGTGGCATTTACAAATA |
|  | R:AGCAGCATAAGTATCCGACATG |
| NjTPS8 | F:ATGGCGGTGGCATTTACAAATA |
|  | R:AGCAGCATAAGTATCCGACATG |
| NjTPS9 | F:AATTTCAAAAATGGGGAAGAAT |
|  | R:GAGACGAAGGCGGTGGCAGATT |

Supplementary Table 4 Candidate Sesquiterpene Synthase Gene qRT-PCR Primers

| Gene | Primer |
| --- | --- |
| NjTPS1 | QF:ATCGCGGCCGTTCATTTTTC |
|  | QR:AAGGCTCATCCCATCGATCC |
| NjTPS2 | QF:GCCGAGATATCCAGGTGGTG |
|  | QR:TTGAGGTAAGGGAGGACGCT |
| NjTPS3 | QF:TGCTTTTTGTGGACCGTTGG |
|  | QR:TTGAGGTAAGGGAGGACGCT |
| NjTPS4 | QF:AGCAGAAGCTTACTTGGCGT |
|  | QR:TGTCAATCATGCAGAGGCGT |
| NjTPS5 | QF:ATTCAAGCGTTCGAGTGGGT |
|  | QR:AACGCTTTCTCCGGATGAGT |
| NjTPS6 | QF:CCGCACTATTGTTGGGTTCG |
|  | QR:GCGTCGGTTGAAGAGTGAGA |
| NjTPS7 | QF:ACGGAGAGGAAAGCGTCAAG |
|  | QR:TGGCTCCATCCATCCAATCG |
| NjTPS8 | QF:ACGGAGAGGAAAGCGTCAAG |
|  | QR:TGGCTCCATCCATCCAATCG |
| NjTPS9 | QF:ATACGGGGCAAGCCAAGAAA |
|  | QR:CGCTTGAGTGAGGTGACGAT |
| GAPDH | QF:GCTGCAAAGGCTGTTGGAAA |
|  | QR:CTCGGTTGCTGTAACCCCAT |

Each gene sequence used for constructing the phylogenetic tree(Figure 5)

>NjTPS1

MGEKSRMSELKFDWHVLISYFLIAAVHFSPNEAEARAAWTKASFIGTIIDDVFDIDGSKEELLNIIELVEKYARRVLSFIYIPSLRFKTIYKLIILICLYTDRWDEPSSVGYCSNRIEILFSAVGDSVDYFVTKEFITQGRYIKDRGAN

>NjTPS2

MGMLSLYEASYMGANGEDILLQAMEFTKNHLKESLPLMESNLGKQVLQSLELPKNLRMARLEARRYIEEYSNESDHNLALLELAKLDYNQVQSLHQMELAEISRWWKHLGLVDKLSFARDRPLECFLWTVGILPEPKDSGCRIELAKTIAILLVIDDIFDTHGSYDELVLFTNAIRRWDLNAMEELPEYMKICYMALYNTTNEICYKVLKENGWSVLPYLKRTWIDMIEGFMVEAKWLNNEEVPNLEEYIENGVTTAGSYMALVHIFFLIGEGVNEDNVKLLLNPYPKLFSSAGRILRLWDDLGTSKEEQERGDVASSIQLFMKENNITCEEEARNQIIQIVQNLWKELNGELMAPNALPLPIIKACLNMARASQVVYQHDGDSYFSNVDNYVQSLFYTPIRM

>NjTPS3

MISSSSVRSLCFPKTNIITSKVPSLLINNINVTSNNSSIRACISMSSLPVSKSTSSSTAAPLIRDNGSLLKFITQTPQVEVDESKRIMELVETTRRTLRKASSDPTDKMKLIDSLQRLGLNHHFEEDINVVLQEFANEQKNTNEDLFTTSLRFRLLRHNGYNVTPDIFNKFTEKNGKFKESLSEDTIGILSLYEASYLGAKGEEILSEAIKFSESKLRESAGHVAPQISRQILQSLELPRHLRMARLESRRFIEEDYSKEIGCDLSLLELAKLDFNYVQSLHQMELAEISRWWKQLGLADKLPFARDRPLECFLWTVGLLPEPKHSECRIELAKTIAVLLVIDDIFDTYGSFDQLVLFTNAIRRWDLDAMEELPEYMKICYMALYNTTNEICYKVLKENGWSVLPYLKRTVILTYLYFK

>NjTPS4

MGKLQQDWEIVMKKYQRKNGSIFNSPATTAAVLTHHLPDAASLNYIRLLLDKFGNAVPTVYPLDIYVRLCMIDNLERLGIDWHFRDEIQTVLDETYRCWLQGDEQIFTDISTCAIAFRLLRMNGYDVSSDALTQIAEEGNYLNSPGDRNLKGISDELELYKASQIIISPDESSALRKQNLQSSNFLKQMLSDDSYCSDKLSRSISQEVDDALNFPFCASLERMANRRYIEQYNVDTSTIRVLKTSYFSSNIGNKDFLKLAVEDFNKCQSRHREDAAYLARWVIENRLDKLKFVRQKSFGYMSFSAAATSFTPKLSDARMSWAKNALLTTVVDDFFDIGGSMDELLNLIYLVDKWDNVDIESDCCSEHVGIIFSALQRGINEIAELAFVYQERNVTSHIVEIWLDLLKSMLREAEWSRDRYVPSMEEYMENGYVSFALGPILLPALYLVGPKLSDESARSFQLKKLFRLMSNCGRLLNDIQGFKRESKEGKLNSVSLRMMMMMNEGDTDEGIIVNELKMLVESYKEELLRIVIEEKESVLPRECKELFWKMTKVVHQFYLKDDGFTSQHMMKAVNDVIYQPIILEEHQLE

>NjTPS5

MEYKWFNKNYVPAFEEYMQKALVTSGNRLLITFSFLGMDEVATIQAFEWVKSNAKMIVSSNKVLRLIDDIMSHEEEDERGHVATGIECFVKEHGLTREEVIVEFHKRIDDAWKDINEEFITPNNLPIEILTRVLNLTRIGDVVYKYDDGYTHPEKALKDHIISLFVDPVSI

>NjTPS6

MQNIPLLMVHLCLILLYTALLLGSYISLLLVQILRMMFILLVSLLLLLRRFTGQLFFVFRGIFEVQFFRVFFSFPPLPWSCVLTPIPIMEVIPHIANLLLVFVFFWAILLFLGRVRNNLLFLTLQPTRSIVIWCLLRKRLFGYVGCMQIWVYCSLNLLLCIVT

>NjTPS7

MAVAFTNISWWVRRGKRQEPKISNGSTLNSSPDTPLRELDTLKFPLVHGGNMASTNKRVKRKWSSRKERKIDREYDVVLVPSDGGCVSGSESDDSDWSIGWMEPHGTGFHSDDDSDDSFAVLVRCYGRGRGDFEENSRDKYLDPIGHMSDTYAA

>NjTPS8

MAVAFTNISWWVRRGKRQEPKISNGSTLNSSPDTPLRELDTLKFPLVHGGNMASTNKRVKRKWSSRKERKIDREYDVVLVPSDGGCVSGSESDDSDWSIGWMEPHGTGFHSDDDSDDSFAVLVRCYGRGRGDFEENSRDKYLDPIGHMSDTYAA

>NjTPS9

MYNLLRASQIVLPGEKILEDAKKFSYQFLKHKRAANQILDKWIITKDLPGEVNYALDMPWYASLSRVETRYYLEQYGGQDDVWIGKTLYRMSNVNNNTYMELAKLDYNNCQAMHLEEWSRIKQWYEECNLGEYGASQESLVLLAYYLASASIFEPEKSKERIAWAKTTTLMETIMSYFGTKNLSIDQRRSFVRDFKKCSNNLHFTYGRYKTGQGLMGVLLDTVYQFSLEALRTHGRDIHRHLTQAWETWMLTWQEEGDVNRAQAELVVRTINLCAGSCSSEEQLLNMMSHPQYHRLSQLTNKICHRLRL

>TwTPS27

MAPLVVSLTISHFVIQTGSTALHYSALPETRTKHCHSSRPFASINSNSLQMNQRPLTDYRPAIWNPELIDSLNTPYSYQSHGTQLDKLRQDAKRLLSSTSDPCLLLNHVESMQRLGIAYHFQEEIDYLLNTRIQPYSPDDHDLHTTALRFRILRDNNFPISSDVFGKFMSREGKFLDSLSRDVKGLLSLYEASFLGVDGEVILDEAKEFSSKNLRALLGRLESTSIDVAEQVKQSLQIPLFWRMPRVEARNFIDFYQKKDAKSSTLLELAKLDFNLVQSTYQQELKELSKWWENLGFKQKLSFTRDRLMQSYFSTTGITFKPQFSKARIAATKFINIVNTIDDIHDYYGSQDDLKLFDSAVKRWDLAAMEELPDYMKICYFAMYNLVNELAYDVLINQGIDVLPCLREAWTKFCGAAFVESQWCYTGYTPSMDDYLKNCWISIGVHGSLNFARAHQQGSRSPIANTPLHCLEDPLLYWSSVICRLNNDLATFQHESKTGEVVSFVKCYMVEKGVSQEQACDEIRELIKHAWKMLNTERRRSDLPPLMVEMCMDTPKLSQCLYQHGDGFGVAIDLTKDVMSSLIFRQIPI

>IrKSL4

MGIVALILIKAAMSLILSSFPLFRSSRSSPASASLAGSGLPKTTPPKTASLQSHSPMFEETKGRIAKLFKKNEVCISTYDTAWVGMVPSPFSSDQPCFPDSLFWLLDNQCPDGSWAQPHHHSHSHSPSLLNKDVLSSTLASILALHKWGLGQHHIAKGLHFLELNFASATDNSQITPLGFDIVFPAMLDHAADLSLNLRLDPTTLNDLMNRRDLELQRCTENGSAETEVYMAYIGEGMGKLHDWESVMKYQRKNGSLFNSPSTTAAAFIALRNSDCLNYLYSALNKFGSAVPAVYPLDIYSQLCIVDNLERLGISRFFSTEIQSVLDETYRCWLQGDEEIIMDASTCGLAFRTLRMNGYKVTSDSFIKVVQDCFSSPGHMRDVNTTLELYRASELMLYPHEIELEKQNSRLRSLLEQELSGGSIQSSQLNAEVKQALDYPFYAALDRMVKKKTIEHYNIDDSRILKTSFRLPSFGNKDLLSLSVQDYNRCQAIHREELREFDRWFVENRLDELEFARHKSAYYYCYFAAAATFFAPELSDARMSWAKNALMTTMVDDLFDVTGSVEEMKNLIQLVELWDVDVSTECCSHKVQILFSALKRTICEVGDRAHQLQGRSIRSHIIVIWLDLLHSMMKEVEWTRDKFVPTMDEYVSNAHVSFALGPIVLPALYLVGPKLSEEMVNHSEYHNLFKLMSMCGRLMNDIRGYEREHDDGKLNAMSLYIMNNGGEITPEVAILEIKSWNDRHRRDLLRLVLEEKSVIPKACKDLFWHMCSVVHLFYNKDDGFWSQELIEVVNQVIHQPILLNHF

>IrKSL2

MSLLLSNSALVGPKFRSSRISHASASLDIGLQRATSPQNASVATCFEETKGRIAKLFHKNELSVSTYDTAWVAMVPSPTSSEEPCFPACLNWLLENQCHDGSWARPHHHHMLKKDVLSSTLACILALKKWGVGEEQISRGLHFVELNFASATEKGQITPMGFDIIFPAMLDNARGLSLNLQLEPTTLNDLIYKRDLELKRCNQSNSAEKEVYWAHIAEGMGKLQDWESVMKYQRKNGSLFNSPSTTAAAFIALRNSDCLNYLYSAMNKFGSAVPAVYPLDIYSQLCLVDNLERLGISRFFSTEIQSVLDDTYRCWLDGDEEIFMDASTCALAFRTLRMNGYSVTSDSFTKAVQDCFSSSIPSHMRDVNTTLELYRASEIMLYPDEIELEKQHSRLRSLLEHELSSGSIQSSQLNAVVKHALDYPFYAILDRMAKKKTIEHYEFDDTRILKTSFCSPTFGNKDFLSLSVEDYNRCQAIHRKEFRELDRWFKETKLDELKFARQKYTYSYCTAAASFASPELSDARMSWAKNSVLIGIVDDLFDVKGSVEEKQNLIKLVELWDVDVSTQCCSQSVQIIFSALRSTICEIGDKGFKIQGRSITDHIIAIWLDVLYNMMKESEWAENKSVPTIDEYMKISHVSSGLGPVVLPSLYLVGPKLSQEMVNHSEYHSLFKLMSTCCRLLNDIRSYEREVEGGKPNALALYRVSSGGEMMMSKEAAISELERLIERQRRELMRTILEESVIPKCCKEIFGH

>IrKSL5

MSLLLSNSALVGPKFRSSRISHASASLDIGLQRATSPQNASVATCFEETKGRIAKLFHKNELSVSTYDTAWVAMVPSPTSSEEPCFPACLNWLLENQCHDGSWARPHHHHMLKKDVLSSTLACILALKKWGVGEEQINRGLHFVELNFASATEKGQITPMGFDIIFPAMLDNARGLSLNLQLEPTTLNDLIYKRDLELKRCNQSNSAEKEVYWAHIAEGMGKLQDWESVMKYQRKNGSLFNCPSTTAAAFTALRNSDCLNYLRLALEKFGSAVPAVYPLDIYSQLCTVDNLERLGISRYFLTEIQSVLDETYRSWLQGDEEIFMDASTCALAFRTLRMNGYNVTSDPITKILQECFSSSFRGNMTDINTTLEIYRASELILYPEERDLDQHNLRLKTFLEQELSSNGFIQSCQLGRNINAEVNQAIEYPFYAIMDRMAKRKNIENYNIDNTRILKTSYRSPNFGNKDFLSLSVEDFNRCQVIHREELRELERWVIENRLDELKFARSKAAYCYFSAAATIFSPELSDARMSWAKNGVLTTVVDDFFDVGGSVEELKNLIQLVELWDVDVSTQCCSPNVQIIFSALKHTICEIADKGFKLQGRSITDHIISIWLDLLYSMMKETELGIDKSFPTMDEYMSNAYVSFALGPIVLPALYLVGPKLSEEMVNHSEYHTLFKLMSTCGRLLNDIRGYERELKDGKISAVSLYIMNNGGEITTEAAISEMRSWIERDRRELLRLVLEENKSVLPKACKKLFWHMCTVVHLFYRKDDGFTSLDLHGVVNAIINEPIVLNQF

>SmKSL2

MALPLSTCLLFHPKESRSRRFCFSPASAASLKSGLHSATSAKIASMPTCFEQTRGRIAKLFHKDELSVSTYDTAWVAMVPSPTSLEEPCFPDCLNWLLENQCHDGSWARPHHHPLLKKDVLSSTLACILALKKWGVGEEQIKRGLHFLELNFASATDKCQITPMGFDIIFPAMLDYARGFSLNLRLDPTTFNDLMHKRDLELKRSNRNYSSETETYWAYIAEGMGELQNWESVMKYQRRNGSLFNCPSTTAAAFIALRNSDCLNYLHLALKKFGNAVSAVYPLDIYSQLCTVDNLERLGISQYFSTEIQNVLDETYRCWMQGNEEIFMDASTCALAFRTLRLNGYDVTSDPVTKILQECFSSSFRGNMTDINTTLELYRASELVLYPDERDLEKQNLRLKLLLEQELSSGLIQSCQLGRSINVEVNQAIEYPFYAIMDRVAKRKSIEIYNFDNTRILKTSYCSPNFGNEDFHFLSIEDFNRCQAAHREELGELERWVVENRLDELKFARSKSAYCYFSAAATFFAPELLDARLSWAKNGVLTTVIDDFFDVGGSVEELKNLIQLVELWDVDICTECYSHNVQIIFSALRCTICEIGDKAFKLQGRCITNHIIAIWLDLLNSMMRETEWARDNFVPTIDEYMSNAHVSFALGPIVLPALYLVGPKLSEEMVNHSEYHNLFKLMSTCGRLLNDIHGYERELKDGKLNALSLYIINHGGEVSKEAAIWEMKSWIETQRRELLRLVLEGKKSVLPKPCRELFWHMCSVVHLFYSKGDGFTSQDLIQLVNTIIHQPILLNDQTGAGLSKLHG

>TwTPS16

MMSLSHPNCIRHSSLPISALPKSKSEVLTETDATILYFQETKERIKKMFDKTQLSVSAYDTAWVAMLSSPNSRQAPCFPECVNWLLDNQLSDGSWGLPPHHPSLVKDALSSTLACLLALKRWGLGEQQMTKGLQFIESNFTSINDEEQHTPIGFNIIFPGMIETAIDMNLNLPLRSEDINVMLHNRDLELRRNKFEGREAYLAYVSEGMGKLQDWEMVMKYQRKNGSLFNSPSTTAAALSQLGNAGCFHYINSLIAKFGNAVPTVYPSDKYALLCMIESLERLGIDSHFSKEIRDVLEETYRCWLQGDEEIFSDADTCAMAFRILRVHGYEVSSDPLTQYAEHHFSRSFGGHLKDFSTALELFKASQFVIFPEESGLEKQMSWTNQFLKQEFSNGTTRADRFSKYFSIEVHDTLKFPFHANVERLAHRRNIEHHHVDNTRILKTSYCFSNISNADFLQLAEEDFNRCQSIHREELKHLERWVVETKLDRLKFARQKMAYCYFSAAGTCFSPELSDARISWAKNSVLTTVADDFFDIVGSEEELANLVHLLENWDANGSPHYCSEPVEIIFSALRSTICEIGDKALAWQGRSVTHHVIEMWLDLLKSALREAEWARNKVVPTFDEYVENGYVSMALGPIVLPAVYLIGPKVSEEVVRSPEFHNLFKLMSICGRLINDTRTFKRESEAGKLNSVLLHMIHSGSGTTEEEAVEKIRGLIADGRRELLRLVLQEKDSVVPRACKDLFWKMVQVLHLFYMDGDGFSSPDMMLNAVNDLLREPISL

>TwTPS17

MMSLSHPNCIRHSSLPISAALPKSKSELLTETDATILYFQETKERIKKMFDKSELSVSAYDTAWVAMVSSPNSRQAPCFPKCVNWLLDNQLSDGSWGLPPHHPLLVKDALSSTLACLLALKRWGLGEQQMTKGLQFIESNFTSINDEEQHTPIGFNIIFPGMIETAIDMNLNLPLRSEDINVMLHNRDLELRRNKLEGREAYLAYVSEGMGKLQDWEMVMKYQRKNGSLFNSPSTTAAALSHLGNAGCFHYINSLVAKFGNAVPTVYPSDKYVLLYMIESLERLGIDRHFSKEIRDVLEETYRCWLQGDEEIFSDADTCAMAFRILRVHGYEVSSDSLTQYAEHHFSHSFGGHLKDFSTALELFKASQFVIFPEESGLEKQMSWTNQFLKQEFSNGTTCADRFSKYFSIEVHDTLKFPFHANVERLAHRRNIEHHHVDKTRIFKTSYCFSNISNADFLQLAVEDFNSCQSIHHEELKHLERWVVESKLDRLKFARQKTAYCYFSAAGTSFSPELSDARISWAKNSVLTTVVDDFFDIGGSEEELANLVHLLEKWDANGSPHYCSEQVEIIFSALRSTICEIGDKALAWQGRSVTHHVIEIWLDLLKSMLREAKWARNKVVPTFDEYVENGYVSMALGPIVLPAVYLIGPKVSKEVVRSPEFHNLFKLMSICGRLINDTRTFKRESEAGKLNSVLLHMIHSGSGTTEEEAIEKIRGLIADGRRELLRLVLQEKDSVVPRACKDLFWKMVQVLHLFYMDGDGFSSPDMMLNAVNDLLREPISL

>TwTPS18

MMSLSHPNCIRHSSLPISVALPKSKSELLTETDATILYFQETKERIKKMFDKSELSVSAYDTAWVAMVSSPNSRQAPCFPECVNWLLDNQLSDGSWGLPPHHPLLVKDALSSTLACLLALKRWRLGEQQMTKGLQFIESNFTSINDEELHTPIGFNIIFPGMIETAIDMNLNVPLRSEDINVMLHNRDLELRRNKLEGREAYLAYVSEGMGKLQDWEMVMKYQRKNGSLFNSPSTTAAAFSHLGNAGCFHYINSLVAKFGNAVPTVYPSDKYVLLCMIESLERLGIDRHFSKKIRDLLEESYRCWLLGDEEIFSDADTCAMAFRILRVHGYEVSSDPLTQYAEHHFSHSFGGHLKDFSTALELFKASQFVIFPEESGLEKQMSWTNQFLKQEFSNGTTCADRFSKYFSIEVHDTLKFPFHANVERLAHRRNIEHHHVDKTRIFKTSYCFSNISNADFLQLAVEDFNSCQSIHREELKHLERWVVESKLDRLKFARQKTAYCYFSAAGTSFSPELSDARISWAKNSVLTTVVDDFFDIGGSEEELANLVHLLEKWDANGSPHYCSEQVEIIFSALRSTICEIGDKTLAWQGRSVTRHVIEIWLDLLKSMLREAKWARNKVVPTFDEYMENGYVSMALGPIVLPAVYLIGPKISEEVVRSPEFHNLFKLMSICGRLINDTRTCKRESEAGKLNSVLLHMIHSGSGTTEEEAVEKIRGLIVDGRRELLRLVLQEKDSVVPRACKNLFWKMVQVLHLFYMDGDGFSSPDMMLNAVNDLLREPISL

>RcKSL1

MFDKIELSVPYDTAWVAMIPSLNSVQAPFFPECTKWIVDNQLSDGSWGLPHHHPLLIKDTLSSTLACVLALKKWGVGETLVNKGLQFIELNSTSLNDEKQHTPIGFDIIFPAMLEHAKELALNLPLKSDVIDAMLHRRDVDLKSGSGGSNTEGRKAYLAYIAEGIGKFQDWEMVMKYQRKNGSLFNSPSTTAAAFSHLRNADCLQYLQSVLQKYGNAVPTIYPLDVYSRLLMVDILERLGIDRHFRKEIKLVLEETYRYWLQGNEEIFLDCITCAMAFRILRVNGYDVSSDVFTQFTEDHFFDSLGGYLKDTRTVLELYRASQILYPDEPLLEKQNSWTNHFLEKCLSSGSSYADGPRECITEVVHNALNCPYYADLERLTNRRSIENYNVDETRILKASYRCLNTGNQHFLKLAVEDFNLCQLIHQEELQQLGRWVVEKRLNKLKFARQKLGYCYFSAAATLFAPELSDARLSAWAKNGVLTTVVDDLFDVGGSVEELINLIQLIEKWDVDESTHFCSEQVEIIFSALRSTISEIGDKAFTWQGRKVTSHVIKIWLDLLKSMLTETLWTKSKSIPTLDEYMINGYVSFALGPIVLPALFLVGPKLTEEDVRDPELHDLFKAMGTCGRLLNDWRGFQRESKEGKLNAVSLHMIQGNGGVNEEEAIRKIKGLINSQRSELLRLVLREKNSNIPRACKDLFWKMIKVLHLFYLKDDGFTSNEMISTANAVITEPVAFHGP

>RcKSL2

MLLTCTNSLKISSQAKEWESKTLTGMSEQLNKIRIPASDIEGTMTSRVKEMLSKVELSVSSYDTAWVAMVPTLDSSKQPLFPKSLKWIMENQQPDGSWGLDLSHPLLIKDSLSSTLACVLALQKWNVGQQLVHKNLDFVQSNIWAATDEHQRSPIGFDMIFPSMIEYGRDMGLNLSLNQSLVEAMLLKRDLETKRLFYYLKDKPSNLAYVAEGLNTLNDWKEVMKFQRSNGSLFNSPSSTAAALIHLHDGKCFEYLNSLTKKFGNAVPTIYPFDIYARLFVIDSLEKLGIDRYVREDKEKMLDDIYRCWMQGSEEIFSDPTCCAMAFRILRTNGYAISSDALANFDEKESLFYEKDAAKSTLELFKASQTTIFQDEPVLDKINAWTSTYLEKELRDDGTIPDKSLHAEVDYALKHIQANLVRLEHRSYIENYNVDNVSLLKASYRFCNVDDNRDLLTFSFQDYNMCSQMHRKELDYLEGWIKKCGIDQLEYARQTIKYAAFSIASSIFQPKFSDGRISWAQNSVLTTIVDDFFDYGGSMEEELVNLIELVQRWDDHTTIGYKSKEVEILFNAVYSTTNDLADKARILQGRCVKKHMIDSWIFLLKAMLKEAEWARNKIVPTMDDFIPNGYISFALGPIILTSLYLVEPLSEEEAVNSEEYEKLYMVISILGRLINDRVATQSDGAQGKLDNIVTLEVINGKGAITEEEVQEKVARTIDSNRRELLRMVSQTEGSIVPKACKDFFWTMSNVLHLFYMGDDGYSSPTKMMSAVNAVINEPIVLP

>RcKSL3

MLLTSTNTLKISSQRKEWEAKDLTGMFHGQVNGRVKIPASDREGPMMSSSIKEMLNNVELSVSSYDTAWAAMVPALDSSKQPLFPKYLSWVMENQKSDGSWGLDLNHPLLIKDSLSSTLACVLTLQRWNVGQQLVHKNLDFIGSNIWAATDESQHSPTGFNMIFPSMIECGTNMGLNLPLNPSSIEAMLIKRDLETESACSLKGGTSNLAYVAEGLTRLDDWKDVMKYQRSNGSFFNSPSSTAAAFIHLRDDEKCFDYLNSLSKRFENAVPTIYPFDIFTKVSILDILEKLGIDRYISKDKERTLDDLHRCWMKGSDEIFLDPTCSAMAFRLLRTNGYAISSDALSKFEEQKHLYNPKDIKCVLELFKASQMAIFQNEPTLDRINYAWTSTYLKEKLLKGVISDKSLREEVDFVLKHPHARLERIENRKFIESYNVDNVSLLTKTSYRFCNVDERYLLTFACQDFNICQSMHQKELDDLERWVIERRISDLKYARQKVKYAYFAIACRLFQPDFLDARISWVQNSVILTVVDDFFEVGGSSLEALSNLIELVERWDEHLTVGYKSEEVKILFHAIYDTINDLADKAYRKQGRCVKRLHLVDTWLITLKSVLKEAEWVTNKTIVTTMDEYISNGYISAGSGSPIIFSSLYFLEVLSEEEIVNSEEYKNLYMHTSMICRLLNDRVQAKSDGAQGKQNSVSLQVIHGNGIITEEEAVKEVTRMIEYHRRELLRMVVEKTEGSIVPKACKDVFWLMSRILHLFYMSSDDGYSSPTKMIHAADAIINEPIIVSQK

>RcKSL4

MLLGSTNTLRISSHGKEWEGKTLTGMPLGKVNQRVKVPASDSEGAITSKIKEMLSKVGLSVSSYDTAWVAMVPTLDSSKQPLFPKSLNWIMENQQSDGSWGLDLQHPLLIKDSLSSTLACVLALQKWNVGQQLIHKGLDFIQSNIWAAKDEHQHSPIGFDIIFPSMIEYGRDMGLNLSLNQSLVETMLLKRELETKSLKDKPSNLAYVAEGLNRLNDWKEVMKFQRSNGSLFNSPSSTAAALIHLHDGKCFEYLNSLAKQFGNAVPTIYPFDIYARLSIIDTLEKLGIDSYVSEDKERVLDDICRCWMQGSEEIFLDPTCCAMAFRLLRMNGYAISSDALANFDEKEKLLHTKDIKAMLELFKASQLEICEDESALCRIYAWTSNYLKEELVNGEIPDKSLQAEVDHALGHPHASMERKEIKNFIENYNADKVSLLKTSYRFCNANENYLLAFSFRDFNMYQSMHREELDDLERWVKQYGLDKLKYARQTIRSAYFSITSSLFQPNHSDARISWAQNTVLTTVVDDFFDFSGSMEELNLIELIERWDEHTTIGFKSKEVEILFNALYGSVNDLADKAYIVQGRCVKRDLIDIWIILLKTMLKEAEWARDKNVPGMDEYIENGYISFALGPVILISLYLMEPLSEEVVTSKEYDNLFIHASIIGRLLNDRVTAKREFAQGKLNSVSLQVVGSNGAITEEEAKEEVTRIITSHRRELLRMVVQTEGSIVPKSCKNLFWTMSKLLHLFYMSEDGYSSPTKMLSAINAIVNEPIVLP

>OsKSL8

MMLLSSSYSGGQFPGVSPLGTRPKRSTTVVPRPVVTRATAGGVRNNLEVVGNAGTLQGMDIDELRVIVRKQLQGVELSPSSYDTAWVAMVPVQGSRQSPCFPQCVEWILQNQQEDGSWGHSAGPSGEVNKDILLSTLACVLALNIWNVGQDHIRRGLSFIGRNFSVAIDGQCAAPVGFNITFSGMLRLAIGMGLKFPVMETDIDSIFRLREVEFERDAGGTASARKAFMAYVSEGLGREQDWDHVMAYQRKNGSLFNSPSTTAASAIHSCNDRALDYLVSLTSKLGGPVPAIYPDKVYSQLCMVDTLEKMGISSDFACDIRDILDMTYSCWMQDEEEIMLDMATCAKAFRLLRMHGYDVSSEGMARFAERSSFDDSIHAYLNDTKPLLELYKSSQVHFLEEDFILENIGSWSAKLLKQQLSFNKISKSLMPEVEYALKYPFYATVEVLEHKGNIERFNVNGFQRLKSGYCGSGADKEILALAVNKFHYAQSVYQQELRYLESWVAEFRLDELKFARVIPLQSLLSAVVPLFPCELSDARIAWSQNAILTAVVDDLFDGGGSMEEMLNLVALFDKWDDHGEIGFCSSNVEIMFNAVYNTTKRIGAKAALVQKRCVIDHIAEQWQVMVRAMLTEAEWAAGKHIPATMGEYMSVAEPSFALGPIVPVSAYLLGEELPEEAVRSPEYGRLLGLASAVGRLLNDVMTYEKEMGTGKLNSVVLLQPLAAGG AASRGGGGAPAPAPASVEAARAEVRRAIQASWRDLHGLVFGSGGGSSSSIIPRPCREVFWHTGK VASVFYQEGDGYARKAMRSMANAVILEPLHLQE

>OsKSL1

MQHRKELQARTRDQLQTLELSTSLYDTAWVAMVPLRGSRQHPCFPQCVEWILQNQQDDGSWGTRGFGVAVTRDVLSSTLACVLALKRWNVGQEHIRRGLDFIGRNFSIAMDEQIAAPVGFNITFPGMLSLAMGMDLEFPVRQTDVDRLLHLREIELEREAGDHSYGRKAYMAYVTEGLGNLLEWDEIMMFQRKNGSFFNCPSTTAATLVNHYNDKALQYLNCLVSKFGSAVPTVYPLNIYCQLSWVDALEKMGISQYFVSEIKSILDTTYVSWLERDEEIMLDITTCAMAFRLLRMNGYHVSSVELSPVAEASSFRESLQGYLNDKKSLLIELYKASKVSKSENESILDSIGSWSGSLLKESVSSNGVKKAPIFEEMKYALKFPFYYTTLDRLDHKRRNIERFDAKDSQMLKTEYLLPHANQDILALAVEDFSSSQSIYQDELNYLECWVKDEKLDQLPFARQKLTYCYLSAAATIFPRELSEARIAWAKNGVLTTVVDDFFDLGGSKEELENLIALVEKWDGHQEEFYSEQVRIVFSAIYTTVNQLGAKASALQGRDVTKHLTEIWLCLMRSMMTEAEWQRTKYVPTMEEYMANAVVSFALGPIVLPTLYFVGPKLQEDVRDHEYNELFRLMSTCGRLLNDSQGFERESLEGKLNSVSLLVHHSGGSISIDEAKMKAQKSIDTSRRNLLRLVLGEQGAVPRPCKQLFWKMCKIVHMFYSRTDGFSSPKEMVSAVNAVVKEPLKLKVSDPYGSILSGN

>OsKSL10

MLPSSICSMGQIPRTSPHYYGMLPKQMSKGHPPMMVTRAVGGVEKGEVGGNVRSLQVMHSKELQAKIRKQLQRVELSPSLYDTAWVAMVPERSSSQAPCYPQCIEWILQNQHDDGSWGINSSSLSVNKDILLSTLACVVALKKWNAGSYHIKRGLNFVGRNFSVAMDVQNIAPVGFNVTFSGLITLASGMGLQLPVWQTDIDEIFHLRKIELERDSGGTISARKAFMAYVAEGFGSLQDWDQVMAYQRKNGSLLFNSPSTTAAAAIHTFNDRTLNYLDSLTNKFGGPVPAMYPQNIYSQLCTVDALERTGISQKFAREIRDILDTTYRSWLHNEEEVMLDIPTCAMAFRLLRTHGYDITSDEMAHFSEQSSFDDSIHGYLNDTKTLLELFKTSQIRFSCEDLVLENIGTWSAKLLKQQLLSNKLSTSAQSEVEYVLKFPLHSTLDRLEHRRNIEQFKVEGSKVLKSGYCGSHSNEEILALAVDYFHSSQSVYQQELKYFESWVKQCRLDELKFARVMPLIVHFSSAATIFAPELADARMVLSQTCMLITVYDDFFDCPEISREEKENYIALIEKWDNHAEIGFCSKNVEIVFYAVYNTYKQIGEKAALKQNRSIMDQLVEDLVSSAKAMMVEADWTATKYIPATMEEYMSNAEVSGAFASFVCPPLYFLGLKLSEEDVKSHEYTQLLKLTNVIGRLQNDSQTYRKEILAGKVNSVLLRALTDSGNTSPESIEAAKEIVNRDAESSM VEMRSLVFSEGGPIPRPCKDRFWEMCKIVFYFYSEDDAYRTPKETMSSARAVILDPLRLIPPPSCPETLSS

>OsKSL11

MMLLSSSYSGGQFPGVSPLGTRPKRSTTVVPLPVVTRATAGGVRNNLEVVGNAGTLQGMDIDELRVIVRKQLQGVELSPSSYDTAWVAMVPVQGSPQSPCFPQCVEWILQNQQEDGSWGHSAGPSGEVNKDILLSTLACVLALNTWNVGQDHIRRGLSFIGRNFSVAIDGQCAAPVGFNITFSGMLHLAIGMGLKFPVMETDIDSIFRLREVEFERDAGGTASARKAFMAYVSEGLGREQDWDHVMAYQRKNGSLLFNSPSTTAASAIHSCNDRALDYLVSLTSKLGGPVPAIHPDKVYSQLCMVDTLEKMGISSDFACDIRDILDMTYSCWMQDEEEIMLDMATCAKAFRLLRMHGYDVSSEGMARFAERSSFDDSIHAYLNDTKPLLELYKSSQLHFLEEDLILENISSWSAKLLKQQLSSNKIMKSLMPEVEYALKYPLYSTVDALEHRGNIERFNVNGFQRPKSGYCGSGADKEILALAVDKFHYNQSVYQQELRYLESWVAEFGLDELKFARVIPLQSLLSALVPLFPAELSDARIAFSQNCMLTTMVDDFFDGGGSMEEMVNFVALIDEWDNHGEIGFCSNNVEIMFNAIYNTTKRNCAKAAALVQNRCVMDHIAKQWQVMVRAMKTEAEWAASRHIPATMGEYMSVGEPSFALGPIVPLSAYLLGEELPEEAVRSPEYGQLLRHASAVGRLLNDVMTYEKEVLTWTPNSVLLQALAAARGGGESPTPPSPACAEAARGEVRRAIQASWRDLHRLVFRDDDGSSIVPRACRELFWGTAKVANVFYQEVDGYTPKAMRGMANAVILDPLHLQQ

>OsKSL5

MILPMSSACLGQFLRASPRGMIEQFNRAPPLRVSIRGAAGVEKSLGLGRNAGSQQGMQKNQLQDKIRKQLREVQLSPSSYDTAWVAMVPVQGSHQTPRFPQCIEWILQNQHDDGSWGTNLPGSVVNKDILLCTLACVVALKRWNTGRDHISRGLNFIGKNFWVAMDEQTIAAPVGFNITFSGLLNLATGTGLEFPVMQTDIDGIFHMRKIELERDAYGTASSRRAFMAYVSEGLGSLQDWDQVMAYQRKNRSIFNSPSAAAATVIGHHNDSALCYLDSLVSKLDGPVPVMYPQNAYSQLGMVDTLEKMGISNNFSCEISDILDMIYRLWIHNEEELMLDMGTCAMAFRLLRMHGYDISSDGMAQFVEQSSFDDSIHGYLNDTKALLELYRSSQIRCLEDDLILQDIGSWSARVLQEKISSKMTHKSEMLEVEYALKFPVYATLERLEQKRNIEQFKTKEQLKIEGFKLLKSGYRGAITHDEILALAVDEFHSSQSVYQQELQDLNSWVAQTRLDELKFARLMPSITYFSAAATMFPSELSEARIAWTQNCILTTTVDDFFDGDGSKEEMENLVKLIEKWDGHGEIGFSSECVEILFYAIYNTSKQIAEKAVPLQKRNVVDHIAESWWFTVRGMLTEAEWRMDKYVPTTVEEYMSAAVDSFALGPTITSALFLVGPELSEEVFRSKEYIHLMNLANTIGRLLNDMQTYEKEIKMGKVNSVMLHALSHSGGGRGSPEASMEEAKREMRRVLQGSRCDLLRLVTRDGGVVPPPCRKLFWFMSKVLHFVYMEKDGYFTADGMMASANAVILDPLQVTLLPSGLGTL

>OsKSL6

MMLPMSSACSGGQFPGASPHGIIPKQFSRAPRIRVSIRGAAGVEKSLGLGRNAGSQQGMHKNELHDKIRKQLRDVQLQPSSYDTAWVAMVPVQGSHQTPRFPQSIEVWILQNQYDDGSWGTNLPGLVVNKDILLCTLACVVALKRWNTGRDHISRGLNFIGRNFSVAMDEQTVAPVGFNITFSGLLSLATRTGLELPVMQTDIDGIIHIRKIELERDAYGTASSRRAFMAYVSEGLGNLQDWNQVMAYQRKNGSIFNSPSATAATIIGHHNYSGLAYLDFVTSKFGGPVPVMYPQNAYSQLCMVDTLERMGISESFACEISDILDMTYRLWMHNEEELMLDMRTCAMAFRLLRMHGYDITSDGMAQFVEQSSFDDSIHGYLNDTKALLELYKSSQLRCLEDDLILEEIGSWSARVLLEKISSKMIHSIELPEVEYALKCPVYAILERLEQKRNIEQFKTKEQLKIEGFKLLKSGYRGVIPNDEILALAVDEFHSSQSVYQQELQDLNSWVAHTRLDELKFARLMPSITYFSAAAVLLPSESSEARIAWTQNCILTTTVDDFFDGEGSKEEMENLVKLIEKWDDHGEIGFSSECVEILFYAVYNTSKQIAEKAMPLQKRNAVDHIAESWWFTVRGMLTEAEWRMDKYVPTTVEEYMSAAVDSFAVGPIITSALFLVGPELSEEVFRSEEYIHLMNLANTIGRLLNDMQTYEKEIKMGKVNSVMLHALSHSGGGRGSPEASMEEAKREMRRVLQGCRFELLRLVTRDAGVVPPPCRKLFWLMSKVLHFVYMEKDRYFTAEGMMASANAVILDPLQVTLPPSDSGTL

>OsKSL7

MMLLGSPSSGGYGGKFAGASPAGTTTMAPSAKQPSSRAPPPGITGGRNDLRILSPAAAAAAVGGLEMKKPEAEGIAESLQATHRKLEASIRKQLQGVELSPSPYDTAWVAMVPLRGSSHNPSFPQCVDWILENQWDDGSWSIDGSISTANKDVLSSTLACVLALNKWNVGREHIRRGLSFIGRNFSIAMDDQAVAPIGFGITFPAMLTLANGSGLEVPVRQNDIDSLNHLREMKIQREAGNHSRGRKAMAYLAEGFGNLLEWDEIMMFQRKNGSLFNCPSTSTAGALANYHDDKALQYLQSLVNKFDGVVPTLYPLNIYCQLSMVDALENMGISQYFASEIKSILDMTYSSWLGKDEEIMLDVTTCAMAFRLLRMNGYDVSSDELSHVAGASGFRDSLQGYLNDRKSVLEVYKTSKHSISENDLILDSIGSWSGGSLLKEMLSSNGKGTPGREEIEFALKYFPYSTLERLVHRKNIVLFDAKGSQMLKTACMPVHDSQDFLALAVDDFCISQSNYQNELNYLESWVKDNRLDQLHFARQKITYCYLSGAATTFRPEMGYARTSWARTAWLTAVIDDLFDVGGLEQEQENLLALMEKWEEPGEDEYYSEDVKIVFQALYNTVNEIGAKASALQGHDVTKYLVDVWLHVVRCMKVEAEWQRSQHLPTFEEYMESGMVSLGQGCTVMSALFLIGEKLPEGIVELEEYDELFRLMGTCGRLLNDIRGIEREESDGKMTNGVSLLVHASGGSMSVDEAKTEVMKRIDASRRKLLSLVVSEQEGPIPRPCKQLFWKMCKILHLFYYQTDDGFSSPKEMVSAVDAVINEPLQLRLL

>EpTPS23

MLLASSTSSRFFTKEWEPSNKTFSGSVRAQLSQRVKNIVVTPDQVKESESSGTSLRLKEMLKKVEMPISSYDTAWVAMVPSMEHSRNKPLFPNSLKWVMENQQPDGSWCFDDSNHPWLIKDSLSSTLASVLALKKWNVGQQLIDKGLEYIGSNMWAATDMHQYSPIGFNIIFPSMVEHANKLGLSLSLDHSLFQSMLRNRDMETKSLNGRNMAYVAEGLNGSNNWKEVMKYQRRNGSILNSPATTAAALIHLNDVKCFEYLDSLLTKFQHAVPTLYPFDIYARLCILDELEKLGVDRFVEIEKMLLLDYIYRCWLEGSEEILEDPTCCAMAFRFLRMNGYVVSPDVLQGFEEEEKLFHVKDTKSVLELLKASQLKVSEKEGILDRIYSWATSYLKHQLFNASISDKSLQNEVDYVVKHPHAILRRIENRNYIENYNTKNVSLRKTSFRFVNVDKRSDLLAHSRQDFNKCQIQFKKELAYLSRWEKKYGLDKLKYARQRLEVVYFSIASNLFEPEFSDARLAWTQYAILTTVVDDFFEYAASMDELVNLTNLIERWDEHGSEEFKSKEVEILFYAIYDLVNEDAEKAKKYQGRCIKSHLVHIWIDILKAMLKESEYVRYNIVPTLDEYISNGCTSISFGAILLIPLYFLGKMSEEVVTSKEYQKLYMHISMLGRLLNDRVTSQKDMAQGKLNSVSLRVLHSNGTLTEEEAKEEVDKIIEKHRRELLRMVVQTEGSVVPKACKKLFWMTSKELHLFYMTEDCFTCPTKLLSAVNSTLKDPLLMP

>IrKSL1

MSLAFNLRVIPFSGHTIQSRRGLFPVHESPMITTKPFAAVKCSLTTSTDLMGKIKEKFNGKVHTSLPAITTHSADTPSNLCIIDTLQRLGVDRYFQSEIDSILDDTYRLWQLKKEDIFSDITTHAMAFRLLRVKGYQVSSEELAPYADQERVNLQEIDVPTVIELYRAAQERVTEEDSTLKKLYVWTSTFLKQQLLTDAIPDKKLHEQVDYYLKNYHGILDRMGVRRSLDLYDVGHYKTLKAADGFSNLCNEDFLAFARQDFNISQAQHQKELQQLQRWYSDCRLDTLKFGRDVVRVSNFLTSAMSGDPELSDVRLAFAKHIVLVTRIDDFFDHGGSKEESYKILELVKEWKEKPAGEYGSEEVEILFTAVYNTVNELAEMAHIEQGRSVKDLLIKLWVEILSMFKIELDTWSDDTALTLDEYLSSSWVSIGCRICILISMQFLGVKLTDEMLLSEECTDLCRHVSMVDRLLNDVQTFEKERKENTGNSVSLLLAAHKDERAINEEEAITKAKDMAEYNRRKLMQIVYKTGTIFPRKCKDMFLKVCRIGCYLYSSGDEFTTPQQMMEDMKSLVYEPLTIHPPEANNVVGKKQSCVSN

>IrKSL3

MSTLKLIPFSTSIDKQFSGRTSILGGKCCLQIDGPKTTKKQSKILVEKIRERISNGKVVEISASAYDTAWVAMVPSREMSGRPSFPECLDWIVENQNPDGSWGLNPFLVKDSLSCTLACLLALRKWGLPNHLLHKGIEFIESNISRAATDDENQVAPIGFNIIFPAMISYAKELDLTLTLPPSSLNALLRARDSEMIRREGKWEYVGEGLGDSCNWNQIIQKHQSRNGSLFNSPATTAAAAIHCRDHKCFDYLISVVNKCNGWAPTVYPMDIYARLCMIDTLQRLGIDCHFRVELDAIFDEIYRNWQEREEEIFSDVTCQALAFRLLRVKGYDVSSDGLEEFVEQEGFFNSVSMQHSNVGTVLELYRASQTRINEEENTLQKIHAWTKPFLTQQLLNKTIRHKPLQMQVEYDLKNFYGTVDRFQHRRTIDLYDAQASQILKTAYRCSAIHNEDFIRFSVQNFKICRAEYQKELDEINKWYAYFGMDLLSKGRNACEQAYVVTAGLIADVELSMARISFAQVILLITVFDDVFDRYGTREEALAVIHLIKEILTHYRWKAAPKECSQLVKTTFTALYDTVNETAAKAHALQGFCFKQQIISLWEELLECAVREKESLSGKNVSTLDEYLSFAPVTIGCELCVLTAVHFLGIQVSEEMLTSAEMLTLCWHGNVVCRLLNDLKTYSREREEKTVNSVSVQVGVSEEEAVAKVKEVLEYHRRKVVEMVYQSQGSNVPRECKELVWKTCKVAHCFYGYDGDEFSSPRDIVDDIKAMMFLGLPHLSTH

>IrKSL6

MFSSSLKLKTNPLMDNKIHRSSSDRDFRGSTISSVKCSLNNSEDLIVKVRERVKGKVEISPSAYDTAWVAMVPERDYSGQKPRFPECLDWIVENQNADGSWGVQSSSMLKHSLSCTLACLLPLRKWNVASPQLLRNGVEFIRSSSSAATDKNQISPIGFDIVFPMMIQYANDLNLELLLNQDLVNILFQNREAQLTRNKNLEYVAEGLGSSIDWNKVLMHQRSNGSLFNSPATTAAALIHRHDKKCLEYLNSLLSIYKTWVPTIHPMDVYARLCLVDHLQGLGVDRFVHPEIEVVLQETFRLWQQKDDKIFTDATCRAMAFRLLRMQGYHVTPDELGGYVDEESFFATVSFESSGTDTVLELYKASQVRLPEDDDTLEKLHDWTSKFLKQKLQSKTILDQQLERKVEFNLKNYHGILDAVKHRRNFDLYDIDHRRILKTAYRCPTVYNEDILLLTAQDLMTRQVQNQKELQIMERWLEDCRLDKVSGRNAVLVSYFLNANNFPDPRLSEARLAYAKTVTLITFLDDFFDHHGSREDSLLIMELINKWTEPLTVSYPSDEVEILYSALHATITDTAEKVYAVQGRCIKSLIIELWMEVLTAMLGEMDSCNADTPPDFDEYMAFAPKSLGCSLSILPSLHLMGETISEEMVTSLECFELDKHVSIAIRLLNDQQTFERERKERTTNSVTLLMDADQISEEEAVSRIQKLIEHHTKELLKLVVQKEGSVLPRKCKDIFWNTIKVGYCLYRFSDEFTSPQQMKEDMKLLFHDPVLKTTP

>SmKSL1

MSLAFNPAATAFSGNGARSRRENFPVKHVTVRGFPMITNKSSFAVKCNLTTTDLMGKIAEKFKGEDSNFPAAAAVQPAADMPSNLCIIDTLQRLGVDRYFRSEIDTILEDTYRLWQRKERAIFSDTAIHAMAFRLLRVKGYEVSSEELAPYADQEHVDLQTIEVATVIELYRAAQERTGEDESSLKKLHAWTTTFLKQKLLTNSIPDKKLHKLVEYYLKNXHGILDRMGVRQNLDLYDISYYRTSKAANRFSNLCSEDFLAFARQDFNICQAQHQKELQQLQRWYADCKDLTLKYGRDVVRVANFLTSAIIGDPELSDVRIVFAQHIVLVTRIDDFFDHRGSREESYKILELIKEWKEKPAAEYGSEEVEILFTAVYNTVNELARAHVEQGRSVKDFLIKLWVQILSIFKRELDTWSDDTALTLDDYLSASWVSIGCRCICILMSMQFIGIKLSDEMLLSEECIDLCRHVSMVDRLLNDVQTFEEKRKENTGNSVTLLLAANKDDSSFTEEEAIRIAKEMAECNRRQLMQIVYKTGTIFPRQCKDMFLKVCRIGC YLYASGDEFTSPQQMMEDMKSLVYEPLTIHPLVANNVRGK

>OsCPS1

QANIIEHETPRITKWPNESRDLDDHQQNNEADEEADDELQPLVEQVRSMLSSMEDGAITASAYDTAWVALVPRLDGEGGTQFPAAVRWIVGSQLADGSWGDEALFSAYDRVINTLACVVALTRWSLHHDQCKQGLQFLNLNLWRLAEEEPDTMPIGFEIAFPSLVEAARGLGIDFPYDHPALKGIYANRELKLKRIPKDMMHIVPTSILHSLEGMPGLDWQRLLKLQCSDGSFLFSPSATAYALMQTGDKKCFAYIDRIIKKFDGGVPNVYPVDLFEHIWVVDRLERLGISRYFQREIEQNMDYVNRHWTEDGICWARNSNVKEVDDTAMAFRLLRLHGYNVSPSVFKNFEKDGEFFCFVGQSTQAVTGMYNLNRASQISFPGEDILQRARNFSYEFLREREAQGTLHDKWIISKDLPGEVQYTLDFPWYASLPRVEARTYIGQYGGNDDVWIGKTLYRMPIVNNATYLELAKQDFNRCQALHQHELQGLQKWFIENGLEAFGMTPEDVLRAYFLAAACIFEPNRASERLAWARVSVLANTISRHFYSDMSSMKRMERFMWSSLYEENGNVLGLEGYAKDGILARTLCQLIDLLSQETPPVREGQKCIHNLIRCAWIEWMMQQINMKDGRYDKGRVMHPGSCTVHNKETCLLIAQIVEICAGRIEEAASMINNTEGSWFIQLASSICDSLHAKMLLSQDTKKNETTINQIDKEIELGMQELAQYLLPRVDDRRINNKTKQTFLSIVKSCYYAANCSPHMLDQHISEVIFEQVI

>OsCPS2

MQMQVLTAAASSLPRATLLRPAAAEPWRQSFLQLQARPIQRPGIMLHCKAQLQGQETRERRQLDDDEHARPPQGGDDDVAASTSELPYMIESIKSKLRAARNSLGETTVSAYDTAWIALVNRLDGGGERSPQFPEAIDWIARNQLPDGSWGDAGMFIVQDRLINTLGCVVALATWGVHEEQRARGLAYIQDNLWRLGEDDEEWMMVGFEITFPVLLEKAKNLGLDINYDDPALQDIYAKRQLKLAKIPREALHARPTTLLHSLEGMENLDWERLLQFKCPAGSLHSSPAASAYALSETGDKELLEYLETAINNFDGGAPCTYPVDNFDRLWSVDRLRRLGISRYFTSEIEEYLEYAYRHLSPDGMSYGGLCPVKDIDDTAMAFRLLRLHGYNVSSSVFNHFEKDGEYFCFAGQSSQSLTAMYNSYRASQIVFPGDDDGLEQLRAYCRAFLEERRATGNLRDKWVIANGLPSEVEYALDFPWKASLPRVETRVYLEQYGASEDAWIGKGLYRMTLVNNDLYLEAAKADFTNFQRLSRLEWLSLKRWYIRNNLQAHGVTEQSVLRAYFLAAANIFEPNRAAERLGWARTAILAEAIASHLRQYSANGAADGMTERLISGLASHDWDWRESNDSAARSLLYALDELIDLHAFGNASDSLREAWKQWLMSWTNESQGSTGGDTALLLVRTIEICSGRHGSAEQSLKNSEDYARLEQIASSMCSKLATKILAQNGG SMDNVEGIDQEVDVEMKELIQRVYGSSSNDVSSVTRQTFLDVVKSFCYVAHCSPETIDGHISKVLFEDVN

>OsCPS4

MPVFTASFQCVTLFGQPASAADAQPLLQGQRPFLHLHARRRRPCGPMLISKSPPYPASEETREWEAEGQHEHTDELRETTTMIDGIRTALRSIGEGEISISAYDTSLVALLKRLDGGDGPQFPSTIDWIVQNQLPDGSWGDASFFMMGDRIMSTLACVVALKSWNIHTDKCERGLLFIQENMWRLAHEEEDWMLVGFEIALPSLLDMAKDLDLDIPYDEPALKAIYAERERKLAKIPRDVLHAMPTTLLHSLEGMVDLDWEKLLKLRCLDGSFHCSPASTATAFQQTGDQKCFEYLDGIVKKFNGGVPCIYPLDVYERLWAVDRLTRLGISRHFTSEIEDCLDYIFRNWTPDGLAHTKNCPVKDIDDTAMGFRLLRLYGYQVPCVLKKFEKDGKFFCLHGESNPSSVTPMYNTYRASQLKFPGDDGVLGRAEVFCRSFLQDRRGSNRMKDKWAIAKDIPGEVEYAMDYPWKASLPRIETRLYLDQYGGSGDVWIGKVLHRMTLFCNDLYLKAAKADFSNFQKECRVELNGLRRWYLRSNLERFGGTDPQTTLMTSYFLASANIFEPNRAAERLGWARVALLADAVSSHFRRIGGPKNLTSNLEELISLVPFDDAYSGSLREAWKQWLMAWTAKESQESIEGDTAILLVRAIEIFGGRHVLTGQRPDLWEYSQLEQLTSSICRKLYRRVLAQENGKSTEKVEEIDQQLDLEMQELTRRVLQGCSA INRLTRETFLHVVKSFCYVAYCSPETIDNHIDKVIFQDVI

>IrCPS2

MGIRLLKMHGYDVDPNALKHFKQEDGKFSCYGGQMIESASPIYNYRASQLRFPGEEILEEATKFAYNFLQEKIANNQIQEKWVISEHLIDEIKLGLKMPWYATLPRVEAAYYLQYYAGTGDVWIGKTFYRMPEISNDTYKELAVLDFNRCQAQHQFEWIYMQEWYQSSSVKAFGISKKELLLAYFLAAATIFEPERTQERIMWAKTQIVSRMIKSFLSKENTLSLEQKTTLLIDFGHDINGLNKINSVEKGNGLAGTLLTTFQQLLEEFDRYTTHQLKNAWSQWFVKLQQGEGDGGADAELLANTLNICAGHIAFNEDILSHRDYTTLSSLTTKICQRLTQIQDKKILEIKDGSIKDKELEEEMQALVKLVLEENGGGIDRNIKQTFLSVFKTFYYCAYHHAETTDAHIFKVLFEPVV

>SmCPS2

MTSLFSLNLSRAPAIRRRLQLPAEVHLSEFFAVYAWLNSSTKPSPLTFQIQRKHLSKVTECRVASLDGIQVSEKDTPLRTPNEINKKIEDSIEYVKNLLMTSGDGRISVSPYDTSIAALIKDVKGRNAPQFPSCLEWIAQHQMADGSWGDEFFCIYDRIVNTLACVIALKSWNVHAHMIQKGVAYVNENVHKLKDGNLEHMTSGFEIVVPALVQRAKDLGIQGLPYDHPLINEIAITKEGRLKKIPKDMIYQTPTTLLFSLEGLGDLEWERILQLQSGDGSFLTSPSSTAHAFMQTKDAKCLKFIDNAVKNCNGGVPHTYPVDVFARLWAVDRLQRLGISRFFQQEIKYFLDHVNSVWTENGVFSGRDSQFCDIDDTSMGIRLLKMHGYNVDPNAVEHFKQQDGKFSCYGGQMIESASPIYNLYRAAQLRFPGEEILEEATKFAFNFLQEKIANNQLQEKWVISHHLIDEIKLGLKMPWYATLPRVEAAYYLRYYAGSDDVWIGKVFYRMPEISNDTYKELAVLDFNRCQAQHQFEWIYMQEWYHRSSVGEFGISKKDLLRAYFLAAATVFEPERTQERLAWAKTQLVSKMIASFVSNETTLSLDQRIALVTQLGHNFDGIDEIISAMKDHALAPTLLTTFQQLLDGFNRYTRHQLKNAWSQWFMKLRQGEANGGGDAELLANTLNICAGLIAFNEDVLLHHEYTTLSTLTNKICKRLSQIKDNKALEVVDGSIKDKELEQDMRALVKLALEENGGGGVDRNIKHTFLSVVKTFYYSAYHDDETTDAHIFKVLFEPVV

>SmCPS1

MASLSSTILSRSPAARRRITPASAKLHRPECFATSAWMGSSSKNLSLSYQLNHKKLSVATVDAPQVHDHDGTTVHQGHDAVKNIEDPIEYIRTLLRTTGDGRISVSPYDTAWVAMIKDVEGRDGPQFPSSLEWIVQNQLEDGSWGDQKLFCVYDRLVNTIACVVALRSWNVHAHKVKRGVTYIKENVDKLMEGNEEHMTCGFEVVFPALLQKAKSLGIEDLPYDSPAVQEVYHVREQKLKRIPLEIMHKMPTSLLFSLEGLENLDWDKLLKLQSADGSFLTSPSSTAFAFMQTKDEKCYQFIKNTIDTFNGGAPHTYPVDVFGRLWAIDRLQRLGISRFFEPEIADCLSHIHKFWTDKGVFSGRESEFCDIDDTSMGMRLMRMHGYDVDPNVLRNFKQKDGKFSCYGGQMIESPSPIYNLYRASQLRFPGEEILEDAKRFAYDFLKEKLANNQILDKWVISKHLPDEIKLGLEMPWLATLPRVEAKYYIQYYAGSGDVWIGKTLYRMPEISNDTYHDLAKTDFKRCQAKHQFEWLYMQEWYESCGIEEFGISRKDLLLSYFLATASIFELERTNERIAWAKSQIIAKMITSFFNKEATSEEEKRDLLNELENINGLNDTNGAGREGGAGSIALATLTQFLEGFDRYTRHQLKNAWSVWLTQLQHGEADDAELLTNTLNICAGHIAFREEILAHNEYKALSNLTSKICRQLSFIQSEKEMGVEGEIAAKSSIKSKELEEDMQMLVKLVLEKYGGIDRNIKKAFLAVAKTYYYRAYHAADTIDTHMFKVLFEPVA

>IrCPS1

MASLSTMHLINHSPASRRRIMSAAAAAKLHMPECFTITKSAWMNNTENLTLNYQLNHKKISKVAGINRVATVDAPQVHDQDDSTENQGHDAVNNIEDPIEYIRTLLRTMGDGRISVSPYDTAWVALIKDLNGRDAPQFPSSLEWIVRNQLDDGSWGDDKFFCVYDRLVNTIACVVALRSWNVHDDKLKRGVTYIKENVEKLRDGNVEHMTCGFEVVFTALLQRAKCLGIEDLPYDSPLIQEIYHSRQQKLNRIPMEMMHKVPTSLLFSLEGLENLEWERLLKLQSADGSFLTSPSSTAFAFMQTKDEKCYQFIKNTVETFNGGAPHTYPVDVFGRLWAVDRLQRLGISRFFESEIAECLAHIHKFWTEKGVFSGRESEFCDIDDTSMGIRLLRMHGYDVDPNVLRNFKKDDNFSCYGGQMIESPSPIYNLYRASQLRFPGEEILEDANKFAYNFLQEKLANNQILDKWVISKHLPDEIKLGMEMPWYATLPRVEARYYLQYYAGSGDVWIGKTLYRMPEISNDTYHELAKTDFKRCQAQHQFEWIYMQEWYESCNVEEFGISRKELLLAYFLATASIFELEKTKERIAWAKSQIISKMITSFFNNHNTSSEEKLAFLTDFRNSNGLNNTNMVLATLTQFLEGFNRYTSHQLKNAWGEWLAKLQQGEGDGAADAELLTNTLNICAGHIAFREEILSHNEYTTLSNLTSKICQQLSQIQNEKKMEIEGQMTAETSIKNKELEQDMQTLVKLVLGKSSGINRNIKKTFLAVAKTYYYRAYHDAQTIDTHMFKVLFEPVV

>IrCPS3

MDDNYRIFNKNLITNAARRGTPQVCTGNGGFEPQPFDKEYVLQKNKIEECITKTLQSIGDGRISASAYDTAWIALIKDVINRDLPLFPWSLEWIVSNQLRDGSWGDRDFFVPCDRLLSTLACVVALSTWNVHPNETERGILFVKENISKLEDGDDVHTTLGFEILFPALLERARNLGIEGLPYDDPTTQKICAERDLKLDRISKELIINEVPASLLLILEGLENLNWENILKLQSPDGSFLGSPATTAFVFMETKDESCLSYLKKTVQNFGGGAPSLYPVDILTRLLAVDRLQRLGISRFFHSQIEDCLSYIYRFWTGNGVFSGRDSEFCDVNDTSMAFRLLRLHGYNVSPNVFTNFKKGDEFSVYGDDEVVDSPSTMLNLYRASEVRFAGETILEEAKEFSHNFLQQRQGLPNQVLDNCLISKNLHTEIKYELETPWFASLPRLEARFFIERYNAVDEVCIGKSLYRLPDTNEGTYLELAKLDYNRCQEQHQMEWNHMQQWYEDCNLEEFGISKNDILEAHFLAAASIFEAERSGERVAWVKTQILSHILSTYYFIKQSHQDHKPQLSTEKAHIAGQPGKGFKNVQRIISILFEALTQMKKDALEGSNGDISDDLLHEAWGGWLKKLGEGETEERQEAELIARTINVCGGHILSKQILSHHEYKTLSQLTNQICHNLHNSKMIGKEMENEMQLLVQLVLQESSNGISKAIKQTFLVVAKAFYYRAYFSAKQIENHVSIILFEQLV

>NtCPS2

MQVIITSSHRFFCHHLHQLKSPTSSLSAQKAEFKKHGPRNWLFQTEGSLLYKPVRILNCATSDASYLGNVNEYLESDHSKNSEEKDIQVSRTIQMKGLTEEIKHMLNSMEDGRLNVLAYDTAWVSFIPNTTNNGNDQRPMFPSCLQWIIDNQLSDGSWGEEIVFCIYDRLLNTLVCVIALTLWNTCLHKRNKGVMFIKENLSKLETGVEENMTSGFELVFPTLLEKAQQLDIDIPYDAPVLKDIYARREVKLTRIPKDVIHTIPTTVLFSLEGLRDDLDWQRLLKLQMPDGSFLISPASTAFAFMETNDEKCLAYLQNVVEKSNGGARQYPFDLVTRLWAIDRLQRLGISYYFAEEFKELLNHVFRYWDEENGIFSGRNSNVSDVDDTCMAIRLLRLHGYDVSPDALNNFKDGDQFVCFRGEVDGSPTHMFNLYRCSQVLFPGEKILEEAKNFTYNFLQQCLANNRCLDKWVIAKDIPGEIWYALEFPWYASLPRVEARYYIEQYGGADDIWIGKTLYRMPDVNNNVYLQAAKLDYNRCQSQHRFEWLIMQEWFEKCNFQQFGISKKYLLVSYFLAAASIFEVEKSRERLAWAKSRIICKMITSYYNDEATTWTTRNSLLMEFKVSHDPTRKNGNETKEILVLKNLRQFLRQLSEETFEDLGKDIHHQLQNAWETWLVFLREEKNACQEETELLVRTINLSGGYMTHDEILFDADYENLSNLTNKVCGKLNELQNDKVTGGSKNTNIELD MQALVKLVFGN TSSNINQDIKQTFFAVVKTFY YSAHVSEEIMNFHISKVLFQQV

>CcCLS

MAFTFTSAHLFLPVTENHSHVHNYSIPPGGNWRLWSTAKGGSNKLDIRRLRCSARRTGEPPLAQGSNGGRDGVEAIQRLQTIGDDKIDGGANELGIVVWDLIRDGVDAVKSMFDSMGDGDISISAYDTAWVALVKDVNGGSGPQFPSSLQWIWDNQLPDGSWGDDSEVFGSAYDRLLKTLACVVALKSWNIRPDKCQKGLKFFRDNISGKLEKENVEASAQMLSGFEVVFLSLIEVARRDLDIQIPLHSPVFEDLIAARRNLKFAKIPLDLMHNVPTGSLGNSLEGMTGVELDWEKLLKLQSQDGSFITSPSSGTAFALMQTNDTKCLGYLKFVVQKFNGGAPGQYPVDIFERIWIVDRLQRLGISRYFQLEIKECCLDYAFKHWTQYGSSWARNTGVYDVDLDDTCVMAFRILRLHGYDVSADAEAFRHFEKNGVFFCFGWETTQSVTVNFNLYRATQVAFPGENILKEAKQFSFNFLMKKQAAREFQDKWVILKDFPGELKYALEFPWYASLPRVETRFYVEQYGGDNDVWIGKTLYRMPYINNNVYLELAKLDFNNCQALHRKEWETMQKWFMESKLDEFGVSSKTLLESYFLAAASIFEPERSTERLAWAKTAFLMETIGSYFDDEMNSKDLRKAFVQEFKNIGYERRMEAKGTKWNLIIGILLTTLNHLTEVCGRDINSYLCHSWEKWMMMWEPEGDRYKGAQAELLNSINLSSGRLFSNGDTLSHPNYEKLVTLSGKNKLCHQLGNSRRGNHNEDSDIKDTKIEIAMQELVQLVHQNSSDDISMGLDKQTFFAVVRSFYYAAHCDRGTINSHIVKVLFESVV

>TwTPS3

MVIMSSHQIFSVSCSSYLHAPLLFPGLSSYTTKDKRVCYFDSTRLICRAISKPARTTPEYSGVLQNGLPLIKWREIVEDDIQEQEEPLKVSLENQIRQGVDIVKSMLGSMEDGEISISAYDTAWVALVENIHHPASSPQFPSALQWIANNQLPDGSWGDPYVFLAHDRLINTLACVIALKKWNIHPNKYKKGLSFVKENISKLEKENEEHMPIGFEIAFPSLLEMAKKLGIEIPDDCPALQDIYAKRDLKLTRIPKDIMHNVPTTLLHSLEGLPGLDWEKLVRLQCKDGSFLFSPSSTACALMHTKDGNCFSYLNNMVQKFNGGVPNVYPVDLFEHIWSVDRLLRLGISRFFQPEIKQCLDHVHRYWTKDGICWARNSSVQDIDDTSMGFRLLRLHGYEVSPDVFKQFRKGDEFVCFVGQSNQAITGIYNLYRASQLMFPEEPILVEAKKFARNFLREKRAVNELLDKWIITKDLPGEVGFALDVPWYACLPRVETRLYIEQYGGQDDVWIGKSLYRMPYVNNNIYLELAKLDYNNCQSLHRTEWDNIQEWYEEYNVRGFGVSKRSLLKTYFVATASIFEPERSVERLAWTKTAILLEVIGSYFKNSREERVEFANEFQKFPKTRGYINARRLDGKMATKEVIEMVFAALNHFSLDALVVHGQDITHHLYQSWEKWVLTWQEEGDRREGEAELLVQTINLMAGHTPSQELLYERLFKLTNKVCHQLGRHHHLNKDKQLQQVQDNGGYNNSNPESISKLQIDSDMRELVQLVLNSSDDMDSNIKQTFLTITKSFYYTAFTHPGTVNYHIAKVLFETV

>TwTPS9

MHSLLMKKVIMYSSQTTHVFPSPLHCTIPKSSSFFLDAPVARLHCLSGHGAKKKRLHFDIQQGRNAVSKTHTPDDLYAKQEYSVPEIVKDDDKEEEVVKIKEHVDIIKSMLSSMEDGEISISAYDTAWVALIQDIHNNGAPQFPSSLLWIAENQLPDGSWGDSRVFLAFDRIINTLACVVALKSWNVHPDKCERGISFLKENISMLEKDDSEHMLVGFEFGFPVLLDMARRLGIDVPDDSPFLQEIYVQRDLKLKRIPKDILHNVPTTLLHSLEAIPDLDWTKLLKLQCQDGSLLFSPSSTAMAFINTKDENCLRYLNYVVQRFNGGAPTVYPYDLFEHNWAVDRLQRLGISRFFQPEIRECMSYVYRYWTKDGIFCTRNSRVHDVDDTAMGFRLLRLHGYEVHPDAFRQFKKGCEFICYEGQSHPTVTVMYNLYRASQLMFPEEKILDEAKQFTEKFLGEKRSANKLLDKWIITKDLPGEVGFALDVPWYASLPRVEARFFIQHYGGEDDVWLDKALYRMPYVNNNVYLELAKLDYNYCQALHRTEWGRIQKWYEECKPRDFGISRECLLRAYFMAAASIFEPERSMERLAWAKTAILLEIIVSYFSEVGNSTEQRIAFTTEFSIRASPMGGYINGRKLDKIGTTQELIQMLLATIDQFSQDAFAAYGHDITRHLHNSWKMWLLKWQEEGDRWLGEAELLIQTINLMADHKIAEKLFMGHTNYEQLFSLTNKVCYSLGHHELQNNRELEHDMQRLVQLVLTNSSDGIDSDIKKTFLAVAKRFYYTAFVDPETVNVHIAKVLFERVD

>TwTPS10

MFMSSSSSSHARRPQLSSFSYLHPPLPFPGLSFSSTRDKRVNFDSTRIISIAKSKPARTTPEYSDVLQTGLPLIVEDDIQEQEEPLEVSLENQIRQGVDIVKSMLGSIEDGEISISAYDTAWVALVENIHHPGSPQFPSTLQWIANNQLPDSSWGDPDMFLTHDRLINTLACVIALKKWNIHPRKCKRGLSFVKENISKLAKEDEEHMLIGFEIAFPSLLEMAKKLGIEIPDDCPAMQDIYTKKDLKLTRIPRDIMHNVPTTLLYSLEGLPSLDWEKLVKLQCQDGSFLFSPSSTACALMHTKDGNCFSYLNNLVHKFNGGVPNVYPVDLFEHIWSVDRLLRLGISRFFRPEIKECLEYVHRYWTKDGICWARNSNVQDIDDTSMGFRLLRLHGYEVSPDVFKQFKKGNEFVCVVGQSDQAITGIYNLYRASQLMFPKETILHGAKEFAGNFLRKKRTANELLDKWIITKDLPGEVGFALDVPWYACLPRVETRLYIEQYGGQDDVWIGKTLYRMPYVNNNVYLELAKLDYNNCQSLHRIEWDNIQKWYEEYNVGGFGVSKRGLLKTYFVATASIFEPERSVERLAWAKTAILVETIRSYFGNSREERIAFPNEFQKAKTRGYINGRRLDGKQATKGLIEMVFATLNHLSQDALVVHGQDITPHLYQSWEKWVLTWQEGGDRGEGEAELLVQTINLMAGHTHSQEEELLYERLFKLTNTVCHQLGHYHHLNKDKQPQQVQDNGGYNNSNPESISKLQIESDMRELVQLVLNSSDGMDSNIKQTFLTVTKSFYYTAFTHPGTVNYHIAKVLFERVV

>TwTPS21

MFMSSSSSSHARRPQLSSFSYLHPPLPFPGLSFFNTRDKRVNFDSTRIICIAKSKPARTTPEYSDVLQTGLPLIVEDDIQEQEEPLEVSLENQIRQGVDIVKSMLGSMEDGETSISAYDTAWVALVENIHHPGSPQFPSSLQWIANNQLPDGSWGDPDVFLAHDRLINTLACVIALKKWNIHPHKCKRGLSFVKENISKLEKENEEHMLIGFEIAFPSLLEMAKKLGIEIPDDSPALQDIYTKRDLKLTRIPKDKMHNVPTTLLHSLEGLPDLDWEKLVKLQFQNGSFLFSPSSTAFAFMHTKDGNCLSYLNDLVHKFNGGVPTAYPVDLFEHIWSVDRLQRLGISRFFHPEIKECLGYVHRYWTKDGICWARNSRVQDIDDTAMGFRLLRLHGYEVSPDVFKQFRKGDEFVCFMGQSNQAITGIYNLYRASQMMFPEETILEEAKKFSVNFLREKRAASELLDKWIITKDLPNEVGFALDVPWYACLPRVETRLYIEQYGGQDDVWIGKTLYRMPYVNNNVYLELAKLDYNNCQSLHRIEWDNIQKWYEGYNLGGFGVNKRSLLRTYFLATSNIFEPERSVERLTWAKTAILVQAIASYFENSREERIEFANEFQKFPNTRGYINGRRLDVKQATKGLIEMVFATLNQFSLDALVVHGEDITHHLYQSWEKWVLTWQEGGDRREGEAELLVQTINLMAGHTHSQEEELYERLFKLTNTVCHQLGHYHHLNKDKQPQQVEDNGGYNNSNPESISKLQIESDMRELVQLVLNSSDGMDSNIKQTFLAVTKSFYYTAFTHPGTVNYHIAKVLFERVV

>TwTPS14

MFMSSSSSSHARRPQLSSFSYLHPPLPFPGLSFFNTRDKRVNFDSTRIICIAKSKPARTTPEYSDVLQTGLPLIVEDDIQEQEEPLEVSLENQIRQGVDIVKSMLGSMEDGETSISAYDTAWVALVENIHHPGSPQFPSSLQWIANNQLPDGSWGDPDVFLAHDRLINTLACVIALKKWNIHPHKCKRGLSFVKENISKLEKENEEHMLIGFEIAFPSLLEMAKKLGIEIPDDSPALQDIYTKRDLKLTRIPKDIMHNVPTTLLYSLEGLPSLDWEKLVKLQCTDGSFLFSPSSTACALMHTKDGNCFSYINNLVHKFNGGVPTVYPVDLFEHIWCVDRLQRLGISRFFHPEIKECLGYVHRYWTKDGICWARNSRVQDIDDTAMGFRLLRLHGYEVSPDVFKQFRKGDEFVCFMGQSNQAITGIYNLYRASQMMFPEETILEEAKKFSVNFLREKRAASELLDKWIITKDLPNEVGFALDVPWYACLPRVETRLYIEQYGGQDDVWIGKTLYRMPYVNNNVYLELAKLDYNNCQSLHRIEWDNIQKWYEGYNLGGFGVNKRSLLRTYFLATSNIFEPERSVERLTWAKTAILVQAIASYFENSREERIEFANEFQKFPNTRGYINGRRLDVKQATKGLIEMVFATLNQFSLDALVVHGEDITHHLYQSWEKWVLTWQEGGDRREGEAELLVQTINLMAGHTHSQEEELYERLFKLTNTVCHQLGHYHHLNKDKQPQQVEDNGGYNNSNPESISKLQIESDMRELVQLVLNSSDGMDSNIKQTFLAVTKSFYYTAFTHPGTVNYHIAKVLFERVV

>CmCPS1

MKALSLSRPFPCSSDATKLSSRPPPPPPSVGSCSFKVESIRSSRIIKCNAISKPPTQEYSDVLQSGVPVIKWQQFVEDDIESETTAHVLISKEIEERVNRIKSMLSSMDDGDISISAYDTAWVALIPRVLDGVKTPLFPSSLEWIAQNQLPDGSWGDSGIFSAHDRILSTLACVLALNSWKLHPDKSEKGMVFLNKNISKLEDENAEHMLIGFEVAFPSLMEFAKRLNLQVPTDSPVLQEINHRRSIKLTRIPKEIMHKVPTTLLHSLEGMEGMEGLDWGMLLKLQAPDGSFLKSPASTAFAFMKTNNSNCFKYLESVVSRFNGGVPNVYPVDLFEHIWAVDRLQRLGVSRFFHPEIVESVDYLRRHWTDKGICWARDVEFYDIDDTAMGFKLLRLFGHEVSAEVFKNFEKDGEFVCIAGQSTQAVTGMFNLYRASDQVMFPGEKILEDAKQFSYKFLREKQAADELLDKWIITKDLPGEVGYALDVPWFASLPRVETRYFIEQYGGENDVWIGKTLYRMFKVNNDTYLELAKLDYNKCQLLHQNEWVDIQKWYTENNLRDYGMRRTSLLFSYFGAACSIFEPERAKERLAWTKTAALVGAIESHFKDANADQRRAFIQQFINFDAIDQAYDTNAWRAGNVQQKGGGQGGLVGILLRTLTSISLDILVSHGFDITHHLHQAWEKWLFKWQEDGDVHKEEAELLVGTIILNSGCSTLEDLLSNPQYQKLSYLTNKVCHQLGHFKKHKVTNGGIYKERTENKMPPEIEEDMRKLLQMVIQNSSDGDNDIDSPIKNTFLTVAKSSYYAAYFDPWTINYHIAKVLFERVF

>CmCPS2

MSSSSSLSLSRHCLSSSFSFRLPNLFPPPAPGGCSLRVKDKGAVLESSIRCIIKCNAISKPPTQDYSDVLQSGVPLLKWQQFVEEGIESETAAQVSVWEEIEERVKWIKSMLSSMDDGDISISAYDTAWVALIPKVTEEGVKSPQFPSSLEWIANNQLPDGSWGDAQIFSPHDRIINTLASLVALKSWNLHPQNTRKGVAFFNQNIWKLEEENAEHMPIGFEIAFPSLLEFAKKLKLGIRSDSPALQQINARRRLKLARIPKDIMHKLPTTLLHSLEGMSGLDWEKLLKLQSQDGSFLSSPASTAFALMHTNHPNCFKYLEASVHRFNGGVPNVYPVDLFEHIWAVDRLQRLGISRFFHPHIVECVNNVRTHWSEKGICWARNSEFRDIDDTAMGFRLLRLYGHDVSAEVFKHFEKDGEFVCIAGQSTQAVTGMLNLYRASDQVMFPGEKILEDAKQFASKFLRQKQAANQLLDKWIIAKDLPGEVGYALDVPVWFASLPRVETRLYIQHYGGKNDVWIGKTLYRMFKVNNDTYLELAKLDYNNCQRLHQIEWVDIQKWYTESKLRDYGMRRSSILFSYFGAVCSIFEPERAKERLAWTKTAALVHTIASHYKDANAHQRRAFLQQFTNFHAAQPYDNNAWRSGNMQQKGGEGLVGILLRTLTNISLDILLSHGVDITHHLHQAWQKWVFKWQEDGDVHKEEAELLVQTIILNSGCSTLEDLLSNSQFQKLSNLTNKVCHQLAHFKKHKVNNGNLYKEKTDNKMPPEIEEDIRKLVQLVIQKSSDGDNDIDSPIKQTFLTVAKSVYYAAYFDAWTINYHIAKVLFERVF

>IrCPS5

MACVVALRSWNIHPHKTDKGIWFMKKNMCRIDEENLEHMPIGFEVALPSLIDIAKKLEIDIPTQTRGLQEIYARREIKLKKIPRDIMHQVPTTLLHSLEGMAGLKWEKLLKLQSEDGSFLFSPSSTAFALQQTRDHNCLKYLTNHIHKFNGGVPNVYPVDLFEHLWAVDRLQRLGLSRYFEPEIEECIAYVHRQWTEKGICWARNSQVEDIDDTAMGFRLLRLHGYEVSADVFRHFKSDGGEFFCFKGQSTQAVTGMYNLYRASQLMFPGENILVDAARFSANFLQLKRAKNDLLDKWIITKDLPGEVGYALDVPWYASLPRVETRFYLDQYGGDDDVWIGKTLYRMPYVNNNKYLELAKLDYNNCQALHQQEWQNILKWYRSCSLGEFGMTERSLLQTYYVAAASVFEPEKSQERLAWAKTAILMETITSHFEFQQLSRDQKRAFITEFEHDSILKYTNGGRYKRRSSLVGTLVRTLNHLSLDILLAHGRDIHQPLKNAWCKWLNSWEEGGDAELLVRTLNLMSGGGRRRRWASEELLSSNPKHEQLLKATIGVCDKLRLFLRRKVQGGNGCMNATGMTTVEIESEMRELVKLVVTRSSSEDLDSEIKQNFLTIARSFYYAAYCNQGTINFHIAKVLFEK

>IeCPS1

MLQSMGDGEISISPYDTAWVALVEDDGGRRRQPQFPSSLEWISSNQLADGSWGDAGTFSIFDRILNTLACVVALRSWNIHPHKTDKGIWFMKKNMCRIDEENLEHMPIGFEVALPSLIDIAKKLEIDIPTQTRGLQEIYARREIKLKKIPRDIMHQVPTTLLHSLEGMAGLKWEKLLKLQSQDGSFLFSPSSGTAFALQQTRDHGCLKYLTNHIHKFNGGVPNVYPVDLFEHLWAVDRLQRLGLSRYFQPEIEECIAYVHRQWTEKGICWARNSQVEDIDDTAMGFRLLRLHGYEVSADVFRHFKSDGGEFFCFKGQSTQAVTGMYNLYRASQLMFPGENILVDAARFSANFLQLKRANNDLLDKWIITKDLPGEVGYALDVPWYASLPRVETRFYLDQYGGDDDVWIGKTLYRMPYVNNNKYLELAKLDYNNCQALHQQEWQNIQKWYRSCSLGEFGMTERSLLQTYYVAAASVFEPEKSSQERLAWAKTAILMETISSHFEFQQLSRDQKRAFITEFEHDSILKYTNGGRYKRRSSLVGTLVRTLNHLSLDILLAHGRDIHQPLKNAWCKWLNSWEEGGDAELLLRTLNLMSGGGRRRRWASEELLSSNPKHEQLLKATIGVCDKLRLFQRRKVQGGNGCMNATGITTVEIESEMRELVKLVVTRSSSEDLDSEIKQNFLTIARSFYYAAYCNQGTINFHIAKVLFEKVL

>SmCPS5

MPLASNPVAFLPSSTAHGDLPAAAFSRSSAGCLQLCRPLTPTSSLQCNAISRPRTEEYIDVIQNGLPVIKWHEIVEDDAEKDSPKDKVGELRDAVRSMLRSMGDGEISISPYDTAWVALVADADGDRPQFPSSLHWISTNQLADGSWGDHATFSIFDRIINTLACVVALTSWDLHPDKTHKGILFIKKNIHRLEEENVEHMPIGFEVALPSLIDIAKQLQIDIPSDTRGLREIYARREIKLKKIPSDILHQMPTTLLHSLEGMPGLMWQKLLKLQSEDGSFLFSPSSTAFALQQTKDHNCLKYLTNHLIKFKGGVPNVYPVDLFEHLWAVDRLQRLGVSRYFQPEIEECVAYVYRYWTEKGICWARNSEIQDIDDTAMGFRLLRLHGYEVSADVFKHFESGGEFFCFKGQSTQAVTGMYNLYRAAQLIFPGENILEDAATFSAKFLQQKRANNELLDKWIITKDLPGEVGYALDVPWYASLPRVETRFYLEQYGGEDDVWIGKTLYRMPYVNNNKYLELAKLDYNNCQALHQQEWKDIQKWYRNSSLGEFGLSEGSLVQAYYVAAASIFEPQKSQERLAWAKTAILMQTITSHFHHSAEQKRVFLHEFQHATGGRYKTTRTLVGTLLRTLNQLSLDILLAHGCHIHQPLKNAWHKWIKTWEGGGGGAELLVQTLNLCGGGRRNRWESEELLSSHPKYEQLLKATVGVCDKLRRFQHRKDCNGCMGSDGGIRTLDIEAEMQELVKLVVTKSPGDLDSEIKQNFFMIARSYYYAAYCNPGTINFHIAKVLFERVQ

>SmCPS4

MSFASNATGFRIPLTTCVYPSPILRFNAKVGSGSSYGTTEAQRNMKCVDGIGRSRVVAVAASGRTRDSNPEVESEKMKEMIRWMFRDMDDGEVSVSAYDTAWVALVEDIGGSGGPQFPTSLDWISDNQLDDGSWGDRKFVLYDRILNTLACVVALTTWKLHPHKCEKGLKFIRENIEKLDNEDEELMLVGFEVALPSLIDLAKKLGIEISDDSPCIKNIYAKRDSKLKEIPMDLLHKEPTSLLFSLEGMEGLDWEKLLTLRSEGSFLSSPSSTAYALQHTKDELCLDYLLKPVNKFNGGVPSTYPVDMFEHLWAVDRLQRLGISRYFQVEIGECLDYVYRYWTNEGISWARYTNIKDSDDTSMGFRLLRLHGYDISIDAFKAFEKGGEFWCMAGQMGHAVTGVYNLYRASQLMFPQEHILLDARNFSANFLHHKRLTNAIVDKWIISKDLPAEVGYALDVPFYASLPRLEARFFLEQYGGDDDVWIGKTLYRMLYVNSNTYLELAKLDYKHCQSVHQLEWKSMQKWYTDCNLGEFGLSERSLLLAYYIAASTAFEPEKSGERLAWATTIILVETIASQQLSNEQKREFVNEFENGSTINNRNGGRYKPRSRLVDVLINAITLVAQGRGISQQLSNAWQKWLKTWEGGGHQGEAEARLLIHTLHLSSGLDESSFSHPKYQQLLEVTSKVCHQLRLFQNRKVYDAQGCTSRLVTGTTFQTEAGMQELVKLVFPKTSDDMTSATKQSFFNIARSFYYTAYCHEGAIDSHIDKVLFEKIV

>IrCPS4

MSSSSNVTSLLRPTTAADGVLPRQMVRVNSSCNIWRSKAKVGGINYFNPGNIKCVEEVHKSRQVAVAALKSLEYETEKPTNQDVVSEKMRVLSKRIETMLQNMDEGEISISPYDTAWVALVEDTDGRPQFPTSLEWISNNQLADGSWGDRKFVIYDRILNTLACVVALTTWNMHPHKCNRGLRFIRDNMEKLENEDEELMPIGFEVVFPSLIEAAQKLGIEIPHIDSPCIKKIQAMRDFKLKRIPMELLHKKPTSLLHSLEGMQGLVWEKLLDFRSDGSFLCSPSSTAYALQHTKDELCLQYLLKAVKKFNGGVPNVYPVDMFEHLWCVDRLQRLGICRYFRAQIKEMLDYVYKYWTDKGICWARNTNVQDVDDTAMGFRLLRMHSYDVSTDVFKQFEKAGEFCCFPGQSTHAITGMYNVYRTSQIMFDGEDILADAKNYSATFLHQKRLANELVDKWIITKDLPGEVGYALDVPFFASLPRLEARFFLEQYGGDDDVWIGKTLYRMPYVNSDTYLELAKLDYEKCQAVHQLEWESIQKWYRDWNLVEFGLSERSLLLAYYIAASTVFEPERSRERLAWAITAILVKTIASQRQLPLEQKGEFLGSILENEDGGRLIEFLINTISQLSSEILVAEGRDITQQLSNTWQKWLKTCKEGGDDDLGEAEARLILHTQHLSSGLDESSFSHPKYHQLLEATSKVCGQLRLFQSRKQVDVDLATGTTFQIEAGMQELVKLVFTKSSEDLDSLTKQSFFSIARSFYYTAYCDEGAINSHIDKVLFEKID

>IeCPS2

MSSSSNVTSLPRLTTAGGVFPREMVRVHSSCNILRSKAKVGGINYFNPGNIKCVEVHKSRQVAVAAVKSLEYETEKPTNQDVVSEKMRVLSERIGTMLQNMNEGEISISPYDTAWVALVEDTDGRPQFPTSLEWISNNQLADGSWGDRKFVIYDRILNTLACVVALTTWNMHPHKCNRGLRFIRDNIEKLENENEELMPIGFEVVFPSLIEAAQKLGIEIPHIDSPCIKKIQAMRDFKLKRIPMELLHKKPTSLLHSLEGMQGLVWEKLLDFRSDGSFLCSPSSTAYALQHTKDELCLQYLLKAVKKFNGGVPNVYPVDMFEHLWCVDRLQRLGICRYFRAQIKEMLDYVYKYWTDKGICWARNTNVQDVDDTAMGFRLLRMHGYDVSTDVFKQFEKAGEFCCFPGQSTHAITGMYNVYRTSQIMFDGEDILADAKNYSATFLHQKRLASELVDKWIITKDLPGEVGYALDVPFFASLPRLEARFFLEQYGGDDDVWIGKTLYRMPYVNSDTYLELAKLDYKKCQAVHQLEWKSIQKWYRDCKLGEFGLGEKRLLLAYFLAASTAFEPEKKGERLAWAKTAFLVETIASQQLSHEQKREFPNEFEHGSSLNMENGGRYKTRTRLVEILSNTVSQLSFETLVAEGRDIKQQLSNTWQKWLKTWEEGGNLGEAEAQLLLQTLHLSSGLDESSFSHPKYHQLLEATCKVCNQLRLFQNRKAHD AQGGISDLVIGTTFQIEASMQELVKLVFTKSSEDLDSITKQSFFAIARSFYYTAYCDAGAINSHIYKVLFENID

>AtTPS15

MEVISIFGPKHGSLISLHSSYNLFKVSKVTRFRRTLFPSNHAKVFRFKALTCDNQESNNMFKELPTSEWTHHFHSIQVDVSEMDAIRIEIDALKPKVKNILMSFQGIDSTKKRILMIYMLISLGLACQFEEEIYETLKEGFGKIEEMMANEEDLYTVSIIFWVFRRYGHYISSDFFRRFKGNDGNFKKSLIGDAKGMLSFYEAANMATTKDYILDEALSFTSSHLESLAANGACPPHMSRRIRNALNASQHWNMEMLVAVEYISFYEKEKDHNEMLLKFSKLNFKFLQLQYLQELKVLTKWYKEVDFVSKLPPYFRDRIVENHFFIQTLFVESQHSRARIMMAKYFILLVIQDDTLDRYASLPEAESLVNSLNRWAPDHAMDKQPDYLKFVFKFILDTFEEFEKELRPEGGSFGVCATIEEFKSLVKANLEAEKWALADNMPSFEEYIEVTGVGITAMTTLMGAMMCMGKIVPKEDYKWLKSRPKIIQALAIKGRLMNDMKGYKEDMSRGYAANAVTCYM KQYRVTEQEALKEFEKMVAVANKTVNEEFLTTMGVSRLVL KLAMGVGLMISITYSEDEGYTHPEGKIKEKMTTLFVDQIPL

>AtTPS16

METITVFGPKHGSPLSLPSRTNMCWEMKPSRFPLTSVRGKPAKQVGLKVSASCDRPISKLPPSKWTNYFHSVLVDVSEMDVLEREIEALKPNVREMLMSSKGYDSVKKRSLMIYLLVSLGLAYHFEEEIEKSLKDGFEKIDEIIAGEDDLYTISTIFWVFRTYGYNMSSDVFRRFKEENGKFKESLIEDARGMLSLYEAAHLGTTTDYILDEALDFASNNLVSLAEDGMCPSHLSTHIRNALSISQHWNMEIIVAVQYIRFYEQEVGHDEMLLKFAKLNFNLVQRLYLQEVKILTKWYKDQDIHSKLPPYYRPVVTEMHFFSTATFFEPQFSHARILQTKLFMAELLVDDTCDRYATFSEVESLINSLQRWAPDDAMDTHPDYLKVVFKFILNAFEECEKELRPQGRSYSLEQTKEEYKRFAKSNLDLAKLAQAGNVPSFEEYMEVGKDEIGAFVIVAGSLMGMDNIDAVEAYDFLKSRSKFSQSSAEIVRYLNDLAGFEDDMRRGCVSTGLNCYMNQYGVTETEVFREFRKMVMNTCKIMNEEFLKTTDVPLRVLKTNFSCVRSGFVGYNEGEGVTYPEGKITKYLTSLYVDQI

>AtTPS13

MESQTKFDYESLAFTKLSHSQWTDYFLSVPIDDSELDAITREIDIIKPEVRKLLSSKGDDETSKRKVLLIQSLLSLGLAFHFENEIKDILEDAFRRIDDITGDENDLSTISIMFRVFRTYGHNLPSSVFKRFTGDDGKFERSLTEDAKGILSLYEAAHLGTTTDYILDEALEFTSSHLKSLLVGGMCRPHILRLIRNTLYLPQRWNMEAVIAREYISFYEQEEDHDKMLLRLAKLNFKLLQLHYIKELKTFIKWWMELGLTSKWPSQFRERIVEAWLAGLMMYFEPQFSGGRVIAAKFNYLLTILDDACDHYFSIPELTRLVDCVERWNHDGIHTLEDISRIIFKLALDVFDDIGRGVRSKGCSYYLKEMLEELKILVRANLDLVKWARGNQLPSFEEHVEVGGIALTTYATLMYSFVGMGEAVGKEAYEWVRSRPRLIKSLAAKGRLMDDITDFESDMSNGFAANAINYYMKQFVVTKEEAILECQKMVVDINKIVNEELLKTTTVPRRVLKQALNFGR LLEVLYTKSDDIYNCSEGKLKEYIVTLLIDPIHL

>AtTPS12

MESQTTFKYESLAFTKLSHCQWTDYFLSVPIDESELDVITREIDILKPEVMELLSSQGDDTSKRKVLLIQLLLSLGLAFHFENEIKNILEHAFRKIDDTGDEKDLSTISIMFRVFRTYGHNLPSSVFKRFTGDDGKFQQSLTEDAKGILSLYEAAHLGTTTDYILDEALKFTSSHLKSLLAGGTCRPHILRLIRNTLYLPQRWNMEAVIAREYISFYEQEEDHDKMLLRLAKLNFKLLQLHYIKELKSFIKWWMELGLTSKWPSQFRERIVEAWLAGLMMYFEPQFSGGRVIAAKFNYLLTILDDACDHYFSIHELTRLVACVERWSPDGIDTLEDISRSVFKLMLDVFDDIGKGVRSEGSSYHLKEMLEELNTLVRA NLDLVKWARGIQTAGKEAYEWVRSRPRLIKSLAAKGRLMDDITDFDSDMSNGFAANAINYYMKQFVVTKEEAILECQRMI VDINKTINEELLKTTSVPGRVLKQALNFGR LLELLYTKSDDIYNCSEGKLKEYIVTLLIDPIRL

>AtTPS14

MALIATKISSRSCFVSAYPNNSPTFLISKFPNTVDSLSPANTAKRSILRNVHASVSNPSKQFHNKTSLEYLHELNIKKIKNILSANVDVPSENLEMIDVIQSLGIDLHFRQEIEQTLHMIYKEGLQFNGDLHEIALRFRLRLRQEGHYVQEIIFKNILDKKGGFKDVVKNDVKGLTELFEAELRVEGEETLDGAREFTYSRLNELCSGRESHQKQEIMKSLAQPRHKTVRGLTSKRFTSMIKIAGQEDPEWLQSLLRVAEIDSIRLKSLTQGEMSQTFKWWWTELGLEKDVEKARSQPLKWWHTWSMKILQDPTLTEQRLDLTKPISLVYVIDDIFDVYGEELELTIFTRVVERWDHKGLKTLPKYMRVCFEALDMITTEISMKIYKSHGWNPTYALRQSWASLCKAFLVEAKWFNSGYLPTTEEYMKNGVVSSGVHLVMLHAYILLGEELTKEKVELIESNPGIVSSAATILRLWDDLGSAKDENQDGTDGSYVECYLNEYKGSTVDEARTHVAQKISRAWKRLNRECLNPCPFSRSFSKACLNIARTVPLMYSYDDDQRLPDEYLKSLM

>AtTPS22

MEAARMGFRAKTLPHLGNGTRLPLKTKLSLFPMHLLQNHTTLSRRSTKLNLCVKACSKTSGVESSRPLPHSAPDLWGDHILSVPTENSEFDTLETEIESIKPKVRNMLMSSHKTDKERICLIHLLICLGTFHYFEKEIEEILEQAFRKLDMLFTDEDDLETTAIMFEVFRLYGHKISCDVFDRFKGVDAKFKEHLVSDVRGMLQLYEAAHLATPFETILDEALSFTRYHLESLAGQQATAPHISRHILNALYKPRFLKMEIIAAREYIHFYQKEGHDETLLKFAKLNFNFCQLHYVRELKTLTKWWKDIDLPYKLPYIRDRLLETFIGVMAVYLEPHYSLGRIIATKVSQVIVVMDDTCDAYGTFSEVRSLIDSLERWDPGAIDKLPSCLRIVIQSIVETMEDIEREMKPRGRSSSVQDTVEEIKIMGRAYAEISKWARAGHVPTFDDYIELGLDSSGIRCFAMYSFISMEDCEENQTNAWFKSKPKMLRALSVIFRLTNDIAGFEEEMRRGEVVNGVNCYVKQHNVTKELAVREIKKMIRDNYKIMMEEFLTIKSVSRPILVRCFNIVRLVNLYYEEGDNFTNPNGKLKDLITSLFFHPLPL

>AtTPS19

MEATRMGFGGLSVPLSLTPNLSLIPQRLLDKHNLSLKPVKIHHLVCVRSTKSSDDLETSRPSTYFSPSLWGDHFLSVSLDHAEFVELEREIETMKPLVKDLMSSQSSDKEKIRLIHLLVSLGSSYHFDKEIQDILKHSFTKLDGIIVEEDDLETISIMFEVFRLYGHKMSCDAFDRFRGGDGRFKESLAKDVRGMLQLFEVAHLGTLSEDIMDEALRFTRNHLESLTSGNVSSASPHILKHIQNSLYIPRYCNIEVLVAREYISYYEQEEGYNEILLKFAKLNFNFCQCHYIQEIKTLTKWWKDLDLASKLPYIRDRSVESHLGGLGPYFEPQYSLGRIIVAKTIMIIVVADDTYDAHATIPEATVLTEYFQRLNIGADDKLSGYLRIVLESVFEVMGEIEQEMSPKGRSYSVKQVLERFKIIAKAYKQLTEWARKGHVPTFDEYMKVGLVTAGMGDYAGYCFIGMEDINEKEAFEWLNSNPLLIDALNVLFRIANDVGTYETEINRGEVANGLNCYMKQYGVTKEEASRELRKMYIYNKKVVVEEFMNSHDRVPRQVLLRCLNFARLFDVIYTEGDGYSEPKGKIEHF MTSLYVHPIPLS

>AtTPS18

MDATRTFFGLPNVHNVPLCLTSNLSLFPQRLLQKHTLPLKPAKKHHLVCVRSTKSSDDLEGSRPSTYFSPSLWGDHFLSVSLDRGEFDELEREIETMKPLVKDMLMSSQSSDKEKIRLIHLLVSLGSSYHFDKEIQDILKHSFTKLDDIIVGEDDLETISIMFEVFRLYGHKMSCDAFDRFRGEDGRFKESLAKDVRGMLQLFEVAHLGTPSEDIMDEASSFAQNHLDSWIGGNVSGATPHLLKHIQNSLYIPRYCNIEVLVAREYISYYEQEEGHNKILLKFAKLNFNFCQFHYIQELKTLTKWWKDLDLASKLPYIRDRLVESHLGGLGPYFEPHYSLGRIIVAKIIMTMVVVDDTYDAHATVPEVAVLTECLQRLNIGADDKLPDYLRTVLESVFEVMGEIEQEMRPKGRSYGVKQVLERFKNVAKADKQLTEWARTGDVPSFDEYMKVGLVTAGMDGYAGYCFIGMEDVSEKEAFEWLSSNPLIIQALNVMFRLANDVGTYETEINRGEVANGLNCYMKQYGVTKEEASQELRKIYSNNKKVVMEEFMNSHDHVPRQVLLRCLNFARLFDVMYTEGDGYSEPKGKIEHFMTSLYVH

PIPLS

>AtTPS21

MGSEVNRPLADFPANIWEDPLTSFSKSDLGTETFKEKHSTLKEAVKEAFMSSKANPIENIKFIDALCRLGVSYHFEKDIVEQLDKSFDCLDFPQMVRQEGCDLYTVGIIFQVFRQFGFKLSADVFEKFKDENGKFKGHLVTDAYGMLSLYEAAQWGTHGEDIIDEALAFSRSHLEEISSRSSPHLAIRIKNALKHPYHKGISRIETRQYISYYEEEESCDPTLLEFAKIDFNLLQILHREELACVTRWHHEMEFKSKVTYTRHRITEAYLWSLGTYFEPQYSQARVITTMALILFTALDDMYDAYGTMEELELFTDAMDEWLPVVPDEIPIPDSMKFIYNVTVEFYDKLDEELEKEGRSGCGFHLKKSLQKTANGYMQEAKWLKKDYIATFDEYKENAILSSGYYALIAMTFVRMTDVAKLDAFEWLSSHPKIRVASEIISRFTDDISSYEFEHKREHVATGIDCYMQQFGVSKERAVEVMGNIVSDAWKDLNQELMRPHVFPFPLLMRVLNLSRVIDVFYRYQDAYTNPKLLKEHIVSLLIETIPI

>AtTPS27

MATLRISSALIYQNTLTHHFRLRRPHRFVCKSMTKTTPDTTLVELSRRSGNYQPSPWNHCYLLSIENKYASETEVITRDVLKKKVKSMLDDEKKSRLEQLELIDDLQKLGVSYHFEIEINDTLTDLHLKMGRNCWKCDKEEDLHATSLEFRLLRQHGFDVSENIFDVIIDQIESNTFKTNNINGIISLYEASYLSTKSDTKLHKVIRPFATEQIRKFVDDEDTKNIEVREKAYHALEMPYHWRMRRLDTRWYIDAYEKKHDMNLVLIEFAKIDFNIVQAAHQEDLKYVSRWWKDTCLTNQLPFVRDRIVENYFWTVGLIYEPQFGYIRRIMTIVNALVTTIDDIYDIYGTLEELELFTSMVENWDVNRLGELPEYMRLCFLILYNEINGIGCDILKYKKIDVIPYLKKSWADLCRTYLVEAKWYKRGYKPSLEEYMQNAWISISAPTILIHFYCVFSDQISVQNLETLSQHRQHIVRCSATVLRLANDLGTSPTELARGDVLKSVQCYMHETGASEERARDHVHQMISDMWDDMNSETKTACNSSSRSRGFKEAAMNLARMSQCMYQYGDGHGCPEKAKTIDRVQSLLVDPIPLDVNRLG

>AtTPS23

MATLRISSALIYQNTLTHHFRLRRPHRFVCKSMTKTTPDTTLVELSRRSGNYQPSPWNHCYLLSIENKYASETEVITRDVLKKKVKSMLDDEKKSRLEQLELIDDLQKLGVSYHFEIEINDTLTDLHLKMGRNCWKCDKEEDLHATSLEFRLLRQHGFDVSENIFDVIIDQIESNTFKTNNINGIISLYEASYLSTKSDTKLHKVIRPFATEQIRKFVDDEDTKNIEVREKAYHALEMPYHWRMRRLDTRWYIDAYEKKHDMNLVLIEFAKIDFNIVQAAHQEDLKYVSRWWKDTCLTNQLPFVRDRIVENYFWTVGLIYEPQFGYIRRIMTIVNALVTTIDDIYDIYGTLEELELFTSMVENWDVNRLGELPEYMRLCFLILYNEINGIGCDILKYKKIDVIPYLKKSWADLCRTYLVEAKWYKRGYKPSLEEYMQNAWISISAPTILIHFYCVFSDQISVQNLETLSQHRQHIVRCSATVLRLANDLGTSPTELARGDVLKSVQCYMHETGASEERARDHVHQMISDMWDDMNSETKTACNSSSRSRGFKEAAMNLARMSQCMYQYGDGHGCPEKAKTIDRVQSLLVDPIPLDVNRLG

>AtTPS24

MATLCIGSAPIYQNACIHNFRLQRPRRFISKSMTKTMPDANPLDLRRRSGNYQPSSWDHSYLLSIENKYVNEKEVITRHVLKKKVKKMLEEVETKSRLEKLELIDDLQKLGVSYHFEQEINNILTNFHLENGKNIWKCDKEEDLHATALEFRLLRQHGFGVSEDIFDVIIDKIESNTFKSDNITSIITLYEASYLSTKSDTKLHKVIRPFATEQIRNFVDDESETYNIMLREMAIHALEIPYHWRMRRLETRWYIDAYEKKHDMNLFLAEFAKIDFNIVQTAHQEDVKYVSCWWKETGLGSQLHFVRDRIVENYFWTVGMIYEPQFGYIRRIVAIVAALITVIDDIYDIYGTPEELELFTAMVQNWDINRLDELPEYMKLCFLTLFNEINAMGCDVLKCKNIDVIPYFKKSWADLCKAYLVEAKWYKGGYKPSVEEYMQNAWISISAPTMLIHFYCAFSGQISVQILESLVQQQQDVVRCSATVLRLANDLATSPDELARGDVLKSVQCYMHETGVSEEEARTHVQQMISHTWDEMNYEARTAARSSSLLSRRFVETAMNLARMSQCMYQHGDGHGCPDKAKIVDRVQTLLVDPIPLD

>AtTPS31

MEAIRVFGLKLGSKLSIHSQTNAFPAFKLSRFPLTSFPGKHAHLDPLKATTHPLAFDGEENNREFKNLGPSEWGHQFLSAHVDLSEMDALEREIEALKPKVRDMLISSESSKKKILFLYLLVSLGLAYHFEDEIKESLEDGLQKIEEMMASEDDLRFKGDNGKFKECLAKDAKGILSLYEAAHMGTTTDYILDEALSFTLTYMESLAASGTCKINLSRRIRKALDQPQHKNMEIIVAMKYIQFYEEEEDCCDKTLLKFAKLNFKFLQLHYLQELKILSKWYKDQDFKSKLPPYFRDRLVECCHFASLTCFEPKYARARIFLSKIFTVQIFIDDTCDRYASLGEVESLADTIERWDPDDHAMDGLPDYLKSVVKFVFNTFQEFERKCKRSLRINLQVAKWVKAGHLPSFDEYLDVAGLELAISFTFAGILMGMENVCKPEAYEWLKSRDKLVRGVITKVRLLNDIFGYEDDMRRGYVTNSINCYKKQYGVTEEEAIRKLHQIVADGEKMMNEEFLKPINVPYQVPKVVILDTLRAAANVSYEKDDEFTRPGEHLKNCITSIYFDL

>PpCPSKS

MASSTLIQNRSCGVTSSMSSFQIFRGQPLRFPGTRTPAAVQCLKKRRCLRPTESVLESSPGSGSYRIVTGPSGINPSSNGHLQEGSLTHRLPIPMEKSIDNFQSTLYVSDIWSETLQRTECLLQVTENVQMNEWIEEIRMYFRNMTLGEISMSPYDTAWVARVPALDGSHGPQFHRSLQWIIDNQLPDGDWGEPSLFLGYDRVCNTLACVIALKTWGVGAQNVERGIQFLQSNIYKMEEDDANHMPIGFEIVFPAMMEDAKALGLDLPYDATILQQISAEREKKMKKIPMAMVYKYPTTLLHSLEGLHREVDWNKLLQLQSENGSFLYSPASTACALMYTKDVKCFDYLNQLLIKFDHACPNVYPVDLFERLWMVDRLQRLGISRYFEREIRDCLQYVYRYWKDCGIGWASNSSVQDVDDTAMAFRLLRTHGFDVKEDCFRQFFKDGEFFCFAGQSSQAVTGMFNLSRASQTLFPGESLLKKARTFSRNFLRTKHENNECFDKWIITKDLAGEVEYNLTFPWYASLPRLEHRTYLDQYGIDDIWIGKSLYKMPAVTNEVFLKLAKADFNMCQALHKKELEQVIKWNASCQFRDLEFARQKSVECYFAGAATMFEPEMVQARLVWARCCVLTTVLDDYFDHGTPVEELRVFVQAVRTWNPELINGLPEQAKILFMGLYKTVNTIAEEAFMAQKRDVHHHLKHYWDKLITSALKEAEWAESGYVPTFDEYMEVAEISVALEPIVCSTLFFAGHRLDEDVLDSYDYHLVMHLVNRVGRILNDIQGMKREASQGKISSVQIYMEEHPSVPSEAMAIAHLQELVDNSMQQLTYEVLRFTAVPKSCKRIHLNMAKIMHAFYKDTDGFSSLTAMTGFVKKVLFEPVPE


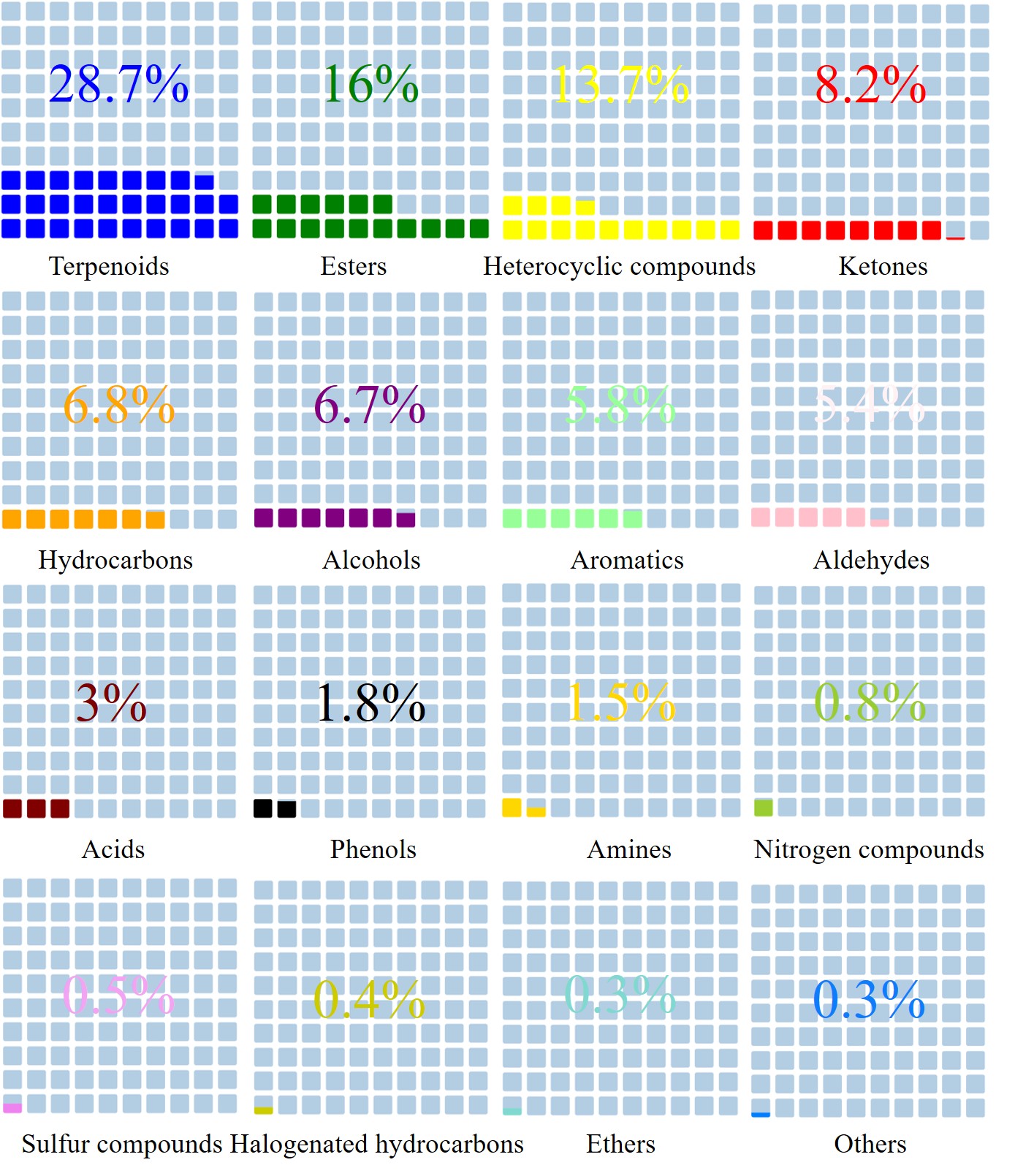


Figure S1 Proportion of different categories of compounds in total volatile metabolites.

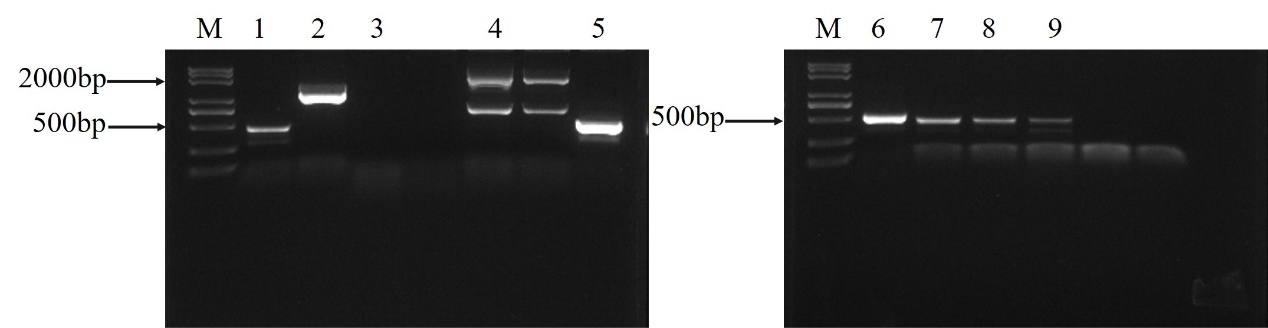


Figure S2 Cloning of Candidate Sesquiterpene Synthase Genes
Note: 1-9 represent NjTPS1, NjTPS2, NjTPS3, NjTPS4, NjTPS5, NjTPS6, NjTPS7, NjTPS8, and NjTPS9, among which NjTPS3 and NjTPS9 failed to be cloned successfully. M is a 5000 bp DNA marker.


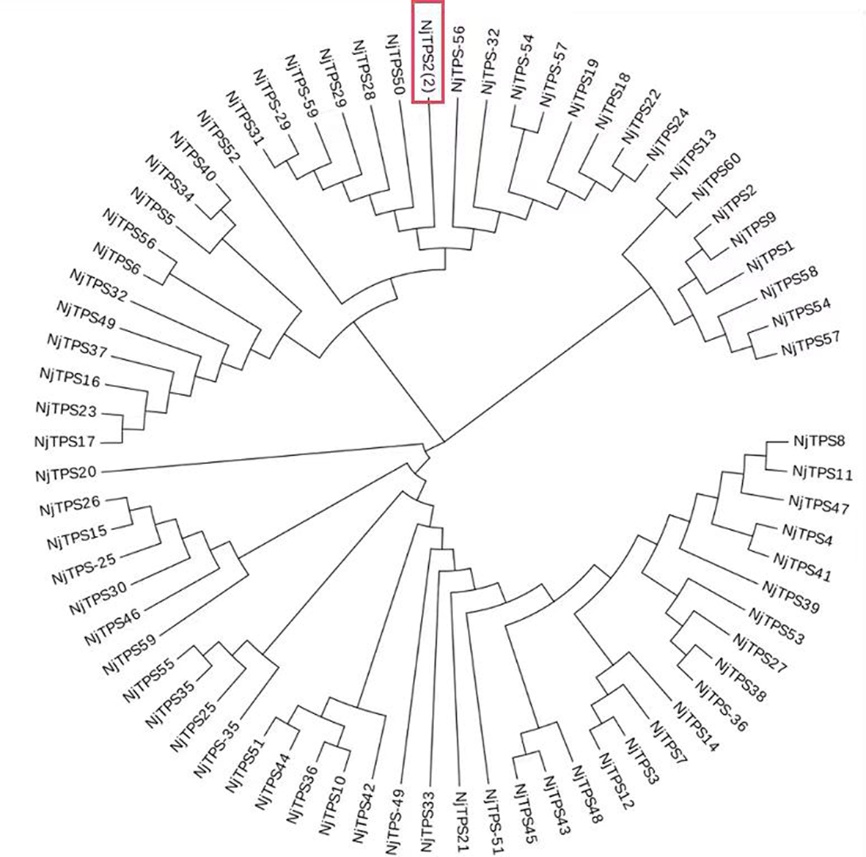


FigureS3: Phylogenetic tree analysis of candidate terpene synthase genes in Tang et al., 2024; Feng et al., 2022

Note: NjTPS2(2) is the gene studied in this paper; NjTPS represents the candidate genes from Feng et al., 2022; Nj-TPS denotes the candidate genes from Tang et al., 2024.
